# Conventional and Molecular Breeding Tools for Accelerating Genetic Gain in Faba Bean (*Vicia Faba* L.)

**DOI:** 10.3389/fpls.2021.744259

**Published:** 2021-10-13

**Authors:** Kedar N. Adhikari, Hamid Khazaei, Lamiae Ghaouti, Fouad Maalouf, Albert Vandenberg, Wolfgang Link, Donal M. O'Sullivan

**Affiliations:** ^1^The University of Sydney, School of Life and Environmental Science, Plant Breeding Institute, Narrabri, NSW, Australia; ^2^World Vegetable Center, Tainan, Taiwan; ^3^Institute of Agronomy and Veterinary Medicine Hassan II, Department of Plant Production, Protection and Biotechnology, Rabat, Morocco; ^4^International Center for Agricultural Research in Dry Areas, Beirut, Lebanon; ^5^Department of Plant Sciences, University of Saskatchewan, Saskatoon, SK, Canada; ^6^Department of Crop Sciences, Georg-August-Universität, Göttingen, Germany; ^7^School of Agriculture, Policy and Development, University of Reading, Reading, United Kingdom

**Keywords:** *Vicia faba*, conventional breeding, synthetic cultivars, marker-assisted selection, genomic selection, biotic and abiotic stresses

## Abstract

Faba bean is a cool-season grain legume crop, which is grown worldwide for food and feed. Despite a decrease in area under faba bean in the past, the interest in growing faba bean is increasing globally due to its high seed protein content and its excellent ecological service. The crop is, however, exposed to diverse biotic and abiotic stresses causing unstable, low grain yield. Although, sources of resistance to main diseases, such as ascochyta blight (*Ascochyta fabae* Speg.), rust (*Uromyces viciae-fabae* (Pers.) Schroet.), chocolate spot (*Botrytis fabae* Sard.) and gall disease (*Physioderma viciae*), have been identified, their resistance is only partial and cannot prevent grain yield losses without agronomical practices. Tightly associated DNA markers for host plant resistance genes are needed to enhance the level of resistance. Less progress has been made for abiotic stresses. Different breeding methods are proposed, but until now line breeding, based on the pedigree method, is the dominant practice in breeding programs. Nonetheless, the low seed multiplication coefficient and the requirement for growing under insect-proof enclosures to avoid outcrossing hampers breeding, along with the lack of tools such as double haploid system and cytoplasmic male sterility. This reduces breeding population size and speed of breeding hence the chances of capturing rare combinations of favorable alleles. Availability and use of the DNA markers such as vicine-convicine (*vc*^−^) and herbicide tolerance in breeding programs have encouraged breeders and given confidence in marker assisted selection. Closely linked QTL for several biotic and abiotic stress tolerance are available and their verification and conversion in breeder friendly platform will enhance the selection process. Recently, genomic selection and speed breeding techniques together with genomics have come within reach to accelerate the genetic gains in faba bean. Advancements in genomic resources with other breeding tools, methods and platforms will enable to accelerate the breeding process for enhancing genetic gain in this species.

## Introduction

Faba bean (*Vicia faba* L.) is a cool-season grain legume cultivated throughout the world for human consumption and animal feed. Its high protein content (25–37%) (Duc et al., 1999; Warsame et al., 2020) makes it a highly valuable grain for both food and feed purposes. Among pulses it occupies sixth place in terms of production after common bean (*Phaseolus vulgaris* L.), chickpea (*Cicer arietinum* L.), field pea (*Pisum sativum* L.), cowpea [*Vigna unguiculata* (L.) Walp], and lentil (*Lens culinaris* Medik.) (FAOSTAT, 2021). Faba bean fits well in cereal-based cropping systems as a rotational crop that enhances soil fertility while breaking the cycle of biotic stresses associated with parasitic weeds and other pathogens. Its nitrogen fixation capacity is one of the highest among legumes, fixing nitrogen even in the presence of high levels of nitrogen in the soil (Herridge et al., 2008) and leaving a significant residue that reduces the need for application of inorganic *N* fertilizer in subsequent crops (Hauggaard-Nielsen et al., 2009).

No extant wild relative capable of producing fertile progeny when crossed with *V. faba* has been found and thus genetic diversity available for breeding purposes is limited to the cultivated genepool (Cubero, 1974; Duc et al., 2010). Recent archaeological findings suggest pre-domestication ancient form of faba bean existed about 14,000 years ago in el-Wad (Mount Carmel, Israel) (Caracuta et al., 2016). Closely related *Vicia* species such as *V. narbonensis* L., *V. galilea* L, and *V. johannis* L. have different numbers of chromosomes (Raina and Rees, 1983), protein profiles compared to *V. faba* (Ladizinsky, 1975), their DNA content is about half of that found in faba bean (Chooi, 1971), and they cannot be crossed with faba bean (Cubero, 1973; Maxted et al., 1991; Duc et al., 2010). Hybrid inter specific embryos were observed between *V. faba* and *V. narbonensis*, and *V. faba* and *V. johannis*, but probably post zygotic barriers stopped their further development and no viable seed could be formed (Ramsay and Pickersgill, 1986; Zenkteler et al., 1998; Wijaya, 2003). Although both *V. johannis* and *V. narbonensis* have resistance to aphids (*Aphis fabae* Scop) and chocolate spot (*Botrytis fabae* Sard), and tolerance to frost (Birch et al., 1985), these traits cannot be transferred to faba bean because of crossing barriers.

However, there is a high degree of genetic diversity in the current gene pool (Duc et al., 2010). Despite having a close genetic similarity within eco-geographical regions, differences exist across wide geographical regions. For example, accessions from China are markedly different from African, European and Asian counterparts (Wang H. et al., 2012). Within China, the spring sown faba beans were different from the winter sown types and they also differed from other Asian accessions which were close to the African and European accessions (Zong et al., 2009). However, Zong et al. (2010) later found the Chinese spring accessions resembled more to the African and European accessions than the Chinese winter types. Although four types of faba bean—*major, minor, equina* and *paucijuga* were described earlier, there is no reproductive barrier among them. The *paucijuga* type is considered as the most primitive extant faba bean lineage (Cubero, 1973; Cubero and Suso, 1981).

The primary center of origin of faba bean is likely to be the Mediterranean basin from where it spread to the Nile Valley, Central and Eastern Asia, and much later South America (Cubero, 1973; Duc et al., 2010). Faba bean is grown as a staple food crop in low rainfall areas in northern Africa (Morocco, Algeria and Tunisia), high rainfall areas in Ethiopia and Eritrea and under irrigation in Sudan and Egypt (north-east Africa) and in the highlands of the South American Andes. These are traditionally faba bean growing areas, but the farming systems have now shifted to monoculture of cereals, resulting in poor soil health, land degradation, increasing greenhouse gases through the use of chemical fertilizer, emergence of new pests and diseases, declining response to farm inputs and yield stagnation (World Bank Group, 2019). As a result, faba bean production in these countries has declined leading to increased cereal mono-cropping and these countries have become importers of faba bean (FAOSTAT, 2021). For example, Egypt, Morocco and Sudan imported faba bean worth US$343 million in 2019 from Australia and Canada (FAOSTAT, 2021). The import in west Asia is about US$69 million.

The global area under faba bean cultivation has almost halved from 5.4 million ha in 1961 to 2.6 million ha in 2019, but its productivity has increased from 0.9 t/ha to 2.1 t/ha in the corresponding years (FAOSTAT, 2021). Reasons for this decline are complex, but include competition from soybean, abandonment in areas where parasitic weeds have become endemic, and yield instability, as the crop is sensitive to many biotic and abiotic stresses. Although significant progress has been made in tackling biotic stresses, research on overcoming abiotic stresses is limited. To date reportedly <50% of potential yield has been achieved in faba bean (Duc, 1997; Mulugeta et al., 2019).

Genomic research in faba bean lags behind other major grain legumes, hampered by its gigantic genome size of 13.1 Gb and lack of investment in underpinning research compared to other crops. Pulses may be considered as research-neglected orphan crops and amongst pulses, faba bean has received comparatively less attention compared to pea, common bean, chickpea, lentil or even cowpea; it is an orphan of the orphans. There is a need to tap on the discoveries made in genetics and genomics research to make significant improvement of this crop. The main breeding objectives are acceleration of genetic gain for improving yield, quality traits and host plant resistance to insect pests and diseases. Traditionally, phenotypic selection has been the major contributor to genetic progress, but with the current advancement of DNA marker technology, phenotypic selection has been enhanced with the use of with marker assisted selection (MAS). DNA markers tightly linked to traits with major effect and high heritability have been discovered so far. However, often traits such as yield are complex and affected by environmental factors and by many other traits, making MAS less effective. Genomic selection based on prediction of breeding values, as a natural extension of MAS, may become available for faba bean as more high throughput dense markers and DNA data sets come available. A recent review by Khazaei et al. (2021) documents the current availability of genomic resources for faba bean. Liu et al. (2019) reported that the efficiency of genomic selection was markedly higher than phenotypic selection and the efficiency would increase further if genomic selection was accompanied by speed breeding.

Gene editing is developing as a new breeding approach in many crops, thus, assisting to accelerate genetic gains. No CRISPR/Cas9 system has been reported for faba bean yet. The absence of an annotated reference genome for this crop poses challenges for the application of CRISPR/Cas gene editing, particularly with the design of specific gRNA-targeted genes of interest (Bhowmik et al., 2021). However, with recent gene discoveries for quality traits such as vicine-convicine (v-c) (Björnsdotter et al., 2021) and seed coat tannins (Gutierrez and Torres, 2019; Gutierrez et al., 2020), this technology may be used to advance our understanding of gene function and accelerate development of new cultivars with reduced anti-nutritional factors. The aim of this review is to highlight species-specific advantages, restrictions and limitations, progress made to date in applied genetics and crossbreeding and what needs to be done to markedly accelerate genetic gains in faba bean.

## Faba Bean Key Breeding Objectives

### Breeding for Resistance to Foliar Diseases

Ascochyta blight (*Ascochyta fabae* Speg.), chocolate spot (*Botrytis fabae* Sard.) and rust [*Uromyces viciae-fabae* (Pers.) Schroet.] are major fungal diseases of faba bean and sources of genetic resistance are available in germplasm collections. These diseases can lower the grain yield by 35 to 90% (Hampton, 1980; Díaz-Ruiz et al., 2009) and 50 to 90% (Gorfu and Yaynu, 2001; Beyene et al., 2016), 30 to 68% (Rashid and Bernier, 1991; Marcellos et al., 1995), respectively. Resistance to these diseases was identified in International Center for Agriculture in the Dry Areas (ICARDA) in the 1980s (Hanounik and Robertson, 1988, 1989) and these genetic resources were later used in faba bean breeding programs globally (Sillero et al., 2010; Temesgen et al., 2015; Adhikari et al., 2016; Maalouf et al., 2016). Despite their use in breeding programs, there is limited understanding of their host-pathogen interactions. A high level of resistance to these diseases has not been found in the germplasm and the resistance is multigenic.

Ascochyta blight is a devastating disease in many countries including Europe, Canada, the Middle East and Oceania (Sudheesh et al., 2019). Sources of resistance to the disease have been reported, but the mode of resistance seems to be complex (Kohpina et al., 2000; Román et al., 2003; Díaz-Ruiz et al., 2009). For example, Kohpina et al. (2000) reported a major dominant gene for resistance in ILB 752, but minor genes in NEB 463. Modern varieties in Australia, such as Farah, Nura, PBA Rana, PBA Samira and PBA Amberley are moderately resistant. In Australia, two pathotypes of the fungus have been identified causing more concern to the breeders. With the availability of closely linked molecular markers (Avila et al., 2004; Kaur et al., 2014; Atienza et al., 2016; Sudheesh et al., 2019), it should be possible to pyramid multiple QTLs and enhance the level of resistance.

Chocolate spot is a serious disease which can cause up to 90% yield losses (Gorfu and Yaynu, 2001; Beyene et al., 2018) in favorable conditions because of its ability to grow on dead tissues in a short latent period (1–3 days). The disease can appear at any growth stage when the environmental conditions are conducive, but if it appears in flowering time, it can cause a complete crop failure. Some resistant lines were reported earlier (Bouhassan et al., 2004), and more lines have been found recently from ICARDA source (Beyene et al., 2018). Many varieties have been developed using ICARDA source of resistance in Ethiopia, Egypt, Sudan, Tunisia, China, Mexico and Australia. For example, Ethiopian researchers have released several high-yielding varieties with partial resistant to chocolate spot, such as Moti, Gebelcho, Gora, Obsie and Walki (Maalouf et al., 2019). Similarly, Australia has released Icarus and PBA Amberley as moderately resistant varieties (Pulse Australia, 2021). Although resistant varieties to the disease have been developed in Australia and Ethiopia, no information is available on whether Australian varieties will be effective against chocolate spot if they were grown in Ethiopia and vice versa.

A moderate level of resistance is available in the germplasm (Bouhassan et al., 2004; Beyene et al., 2018; Khazaei et al., 2018a; Maalouf et al., 2019), but to date no reliable DNA markers have been reported for this disease. Without availability of such markers, chocolate spot has been the most difficult disease to breed mostly because screening for the disease in field condition is unreliable. Faba bean is mostly grown in dry environments as a rain fed crop and the opportunity for its screening can occur only in humid conditions which occur seldom. Therefore, to date, improvement in host plant resistance to this disease has been very slow. An average reduction of 0.27% infection per year over 30 years was reported in Ethiopia (Tolessa et al., 2015) suggesting a coordinated and concentrated effort is needed for breeding resistance to this disease.

Rust is another important disease of faba bean causing 30–68% yield losses (Rashid and Bernier, 1991; Marcellos et al., 1995). Several sources of resistance to rust have been reported (e.g., Rashid and Bernier, 1986, 1991; Avila et al., 2003; Adhikari et al., 2016) but the level of resistance is only partial. At least three resistance genes, *Uvf1, Uvf2* and *Uvf3* have been identified (Avila et al., 2003; Adhikari et al., 2016; Ijaz et al., 2021b). *Uvf2*, located on chromosome III and *Uvf3* on chromosome V, are dominant and independent genes, but their relationship with *Uvf1* is not yet known (Ijaz et al., 2021b). Both genes can be tagged using KASP markers for marker-assisted selection. This should allow pyramiding of genes for enhancing the level of resistance.

Faba bean gall caused by *Physioderma viciae* (You et al., 2021) is a relatively new disease that is currently widespread in Ethiopia. It is not caused by *Olpidium viciae* Kusano as suggested earlier. Recent findings in Ethiopia indicate the availability of partial resistance in existing faba bean germplasm (Yitayih and Azmeraw, 2017; Wondwosen et al., 2019; Kassa et al., 2020). Molecular based research such as identifying quantitative trait loci (QTL) for host plant resistance has not yet been undertaken for faba bean gall.

Host plant resistance breeding is further complicated by the presence of pathogen variability in all three of the major diseases; rust (Herath, 1997; Ijaz et al., 2021a), chocolate spot (Hanounik and Maliha, 1986; Gorfu, 1996), and ascochyta blight (Stoddard et al., 1999). Despite having a moderate level of resistance in selected germplasm, little effort has gone into developing reliable DNA markers that can be used by breeders as part of selection strategies.

#### Viral Diseases

The most prevalent of the many viral diseases than can affect faba bean are Broad bean mottle virus (BBMV), Broad bean stain virus (BBSV), Bean leaf roll virus (BLRV), Bean yellow mosaic virus (BYMV), Alfalfa mosaic virus (AMV), Faba bean necrotic yellows virus (FBNYV) and True broad bean mosaic virus (TBBMV) (Saxena, 1991; Bond et al., 1994; van Leur et al., 2006). Most viral diseases are not host specific and can infect various plant species, thereby allowing them to survive and multiply easily by-passing from one species to another throughout the seasons.

The typical mode of virus disease transmission is through seed and/or insect vectors such as aphids. Non-persistently transmitted viruses, such as BYMV and Pea seed borne mosaic virus (PsbMV) can be transmitted through seed, however, the seed transmission in faba bean is almost negligible in Australia (van Leur et al., 2006). Once a plant is infected by a virus, it cannot be cured, so prevention is the only strategy for controlling these diseases. The major virus affecting faba bean production in North Africa and West Asia is FBNYV which causes up to 90% yield loss in Egypt (Kumari and Makkouk, 2007). In Australia, where virus diseases cause sporadic losses, BYMV, BLRV and PsbMV are the most prevalent viruses. In 2020, however, BYMV caused up to 70% grain yield losses in northern New South Wales (van Leur, pers. comm.), thereby making it a larger risk than fungal diseases that can be controlled by fungicides. Newer cultivars, such as “PBA Nasma” and “PBA Nanu” in Australia are resistant to BLRV, but no effective resistance has been found for BYMV yet.

#### Parasitic Weeds

*Orobanche* and *Phelipanche* (broomrapes) species are root-parasitic plants that can devastate faba bean crops in Mediterranean Europe, West Asia and North Africa, thus resulting a drastic reduction in the crop area (Gressel et al., 2004; Khalil et al., 2004; Maalouf et al., 2011; Rubiales et al., 2016). These parasitic weeds lack chlorophyll and functional roots, and are completely dependent on the host plant. Among the several broomrape species that can infect faba bean, *Orobanche crenata* Forsk. and *O. foetida* Poir. are the most damaging and widespread weeds (Pérez-de-Luque et al., 2010; Rubiales and Fernández-Aparicio, 2012; Rubiales et al., 2014). These weeds are continuing to expand their ecological range to Ethiopia and Sudan forcing farmers to abandon faba bean cultivation in many parts of the country (Abebe et al., 2013).

However, there are some encouraging results with the finding of genotypes resistant to *Orobanche* (Cubero, 1994; Maalouf et al., 2011; Rubiales et al., 2014). The first significant finding of resistance was the identification of the family 402 derived from the cross Rebaya 40/F216 made at ICARDA (Cubero, 1994). Since then, different elite lines with large seed and Orobanche resistance were developed from ICARDA leading to the release of Orobanche resistant cultivars, such as Giza 402, Cairo 843, Misr1, and Misr3 in Egypt, Najah and Chourouk in Tunisia and Ashengie in Ethiopia (Maalouf et al., 2016, 2018). Similarly, Rubiales et al. (2014) found several accessions—V-1268, V-1302, V-1301, V-268, V-231, V-319 and V-1272 along with cultivar Baraca with resistance to both to *O. crenata* and *O. foetida* across many locations. In addition to *Orobanche*, another stem parasitic weed, dodder (*Cuscuta species*) is starting to become a serious problem in faba bean and other legumes in West Asia and North Africa. Although none of the above lines have complete resistance to *Orobanche*, they will certainly help to reduce the burden of parasitic weeds in faba bean. Use of partially resistant cultivars with the application of one to two sub lethal dose of glyphosate at flowering stage is the most practical method to reduce yield loss and weed seed bank over time (Zahran et al., 1980; Rubiales and Fernández-Aparicio, 2012).

### Breeding for Abiotic Stresses

Heat, drought, frost and water logging are major abiotic stresses affecting the faba bean productivity. A comprehensive review on abiotic tolerance for grain legumes has been presented by Toker and Mutlu (2011). While the first two factors affect the crop globally, the latter two are ecologically and geographically specific to local environments. Waterlogging is a problem in limited geographical areas such as the high rainfall regions that are dominated by vertisols in Ethiopia, and the irrigated Nile River basin in Egypt. The effect of frost on vegetative growth has been widely studied in Europe (Arbaoui et al., 2008; Link et al., 2010; Sallam et al., 2015). Research on the effect of frost on the reproductive structures is not reported, as the crop in that region flowers when frost does not occur. However, this could be a future problem as the crop expands to northern latitude in continental climates. Furthermore, tolerance to seedling frost is not related to frost after flowering. Link et al. (2010) identified several accessions with superior frost tolerance, such as Côte d' Or, Hiverna, ILB3187, ILB2999, ILB14, ILB345. Promising winter hardy and frost tolerant faba beans might be found in the Hindu Kush area because materials from these regions are adapted to frost early in the vegetation phase (Olszewski, 1996). Inci and Toker (2011) found three accessions of faba bean from Turkey with freezing tolerance (Supplementary Table 1) and they also noted that wild relatives of faba bean, *viz. V. narbonensis* L. and *V. montbretii* Fisch. et C. A. Mey were more frost tolerant than faba bean. No comparative studies are conducted on frost damage in Australia, but in severe cases the losses can be as high as 60% (Maqbool et al., 2010). Frost is not a severe problem in southern Australia as the crop is acclimatized and hardened due to the much colder environment during the vegetative stage. It is, however, a problem in northern New South Wales and southern Queensland where the daytime temperatures are relatively warm, and night frost occurs, exposing plants to sudden sub-zero temperatures (Maqbool et al., 2010; Alharbi and Adhikari, 2020). Several frost tolerant genotypes at the vegetative stage, such as 11NF010a-2, PBA Warda, PBA Nasma and PBA Nanu were identified in Australia (Alharbi, 2018).

Heat stress in faba bean during the vegetative period can retard plant growth and development, but it is particularly harmful at the reproductive stage, causing reduction in pollen growth and viability that results in significant yield loss (Bishop et al., 2016a). Extreme heat is the major threat to faba bean production in southern Egypt, Sudan, the Ethiopian lowlands, and in northern New South Wales and southern Queensland in Australia. Research conducted under high temperatures (above 35°C) have identified some heat tolerant genotypes, but wider testing for confirmation is still needed (Maalouf et al., 2019).

Low rainfall and variable soil moisture in dry areas are the major reasons for low and unstable grain yield of faba bean, especially where the crop is grown under rain fed conditions in the Mediterranean basin, East and North Africa, West Asia and Australia (Siddique et al., 2001). Drought reduces pollen viability and germination, but recent findings showed the female reproductive tissue was more sensitive to drought than the male part (Muktadir et al., 2021). Development of early maturing cultivars has been a breeding strategy to escape terminal drought. Although genotypic variation for drought tolerance has been documented (Abdelmula et al., 1999; Amede et al., 1999; Link et al., 1999; Khazaei et al., 2013; Muktadir et al., 2021), underlying mechanisms and selection methods for screening germplasm are not fully developed, thereby slowing the progress toward drought-tolerant cultivars. However, some characteristics, such as leaf-level carbon isotope discrimination, stomatal conductance and canopy temperature can be used as selection criteria for drought tolerance in faba bean and can be used for screening large sets of germplasm for drought tolerance under field trials (Khan et al., 2007; Muktadir, 2009).

Soil acidity is among the common problems limiting faba bean production in Ethiopia (Asefa et al., 2010; Jida and Assefa, 2014). It is associated with toxicities of hydrogen ion, aluminum, and manganese, and deficiency of calcium, molybdenum and phosphorus in the soil (Chen et al., 1993). Soil acidity also adversely affects survival, growth and nitrogen-fixation efficiency of Rhizobia (Graham, 1992; Zahran, 1999). Generally, *Rhizobium* strains vary markedly in their acid tolerance and ability to modulate on acid soils and some acid tolerant Rhizobia strains have been identified (Chen et al., 1993; Del Papa et al., 1999; Asefa et al., 2010; Jida and Assefa, 2014), but a higher acid tolerance of the bacteria does not mean a better symbiotic performance under acidic conditions. Therefore, both acid tolerance and symbiotic effectiveness are needed to improve nitrogen fixation, but these traits are not necessarily linked (Howieson et al., 1988). Belachew and Stoddard (2017) identified certain faba bean accessions, mostly from Ethiopia that were tolerant to acid soils (Supplementary Table 1) while Jida and Assefa (2014) identified certain Rhizobia tolerant to highly acidic soils in Ethiopia indicating evolutionary co-existence of symbiosis in faba bean under acid soil. Soil salinity is another significant problem facing agricultural production mostly in semi-arid agriculture systems causing 12-50% yield loss (Farooq et al., 2017). Genotypic variation for salinity tolerance exists in faba bean (Tavakkoli et al., 2012) and several QTL for ionic concentrations for sodium, potassium and chlorine were described by Asif and Paull (2021) for the first time, but these are yet to be verified.

In summary, there is evidence of availability of genetic resources for heat, drought frost, acidic soil and salinity tolerance and there are efficient methods for phenotypically screening against these traits (Stoddard et al., 2006; Zhou et al., 2018). However, there is a lack of efficient genetic methods of integrating these resources into effective breeding programs for developing cultivars with tolerance to these stresses. Major sources for resistance to biotic and abiotic stresses in faba bean are presented in Supplementary Table 1.

## Faba Bean Breeding Methods

**Figure 1:**
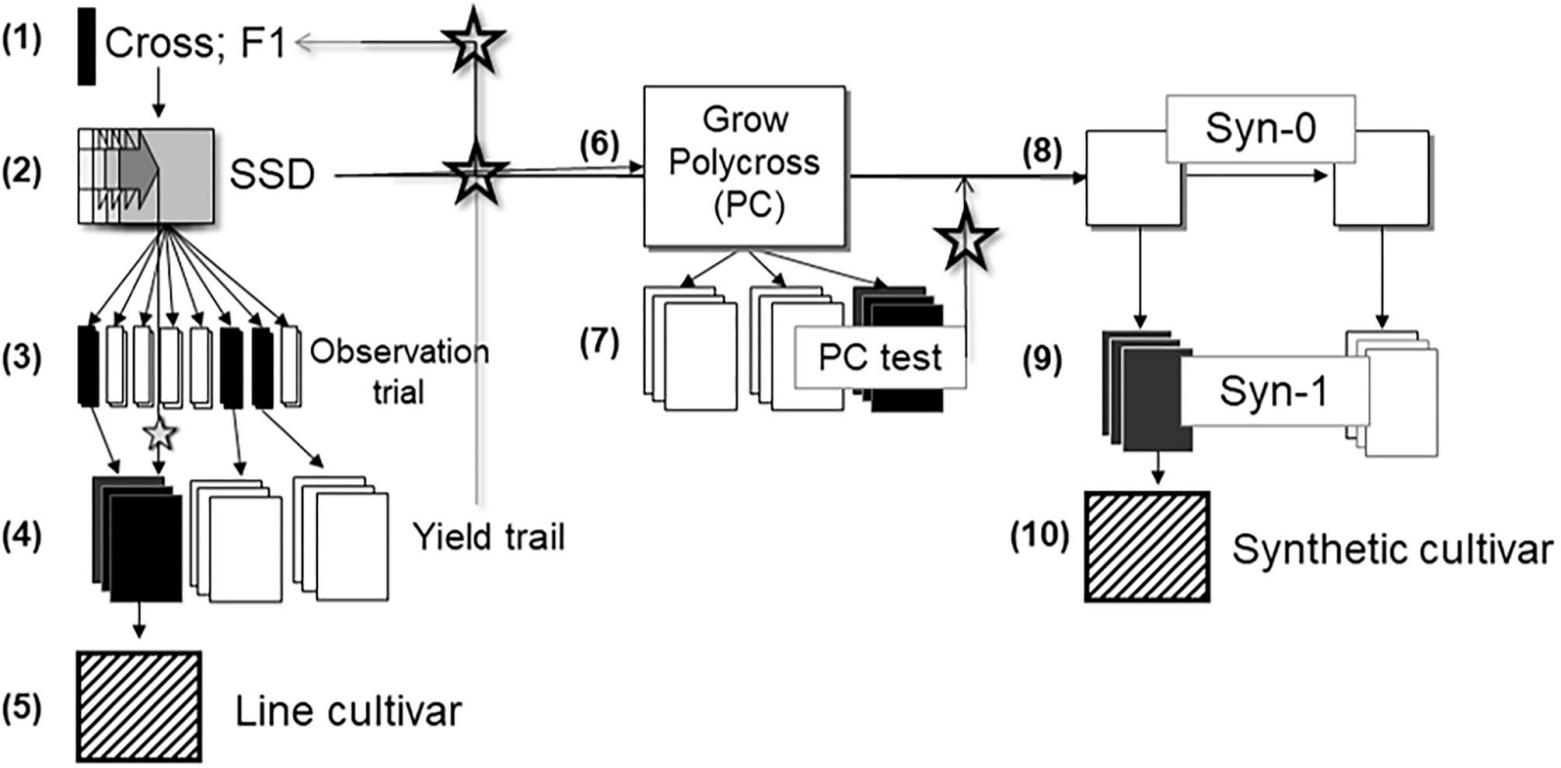
Phenotypic-based breeding scheme in faba bean. (1) Making crosses, (2) Run SSD (single seed descent) until about F7, (3) Multiply SSD F8 individuals in open field and apply phenotypic selection on *per se* performance, (4) Test open pollinated offspring in replicated yield trials and identify best SSD lines as new crossing parental lines, (5) Identify and multiply the best SSD line(s) and release as a line cultivar or take the best lines as parents for a polycross, (6) Grow a polycross from selected SSD lines, (7) Run yield trial (PC test) and identify offspring of best SSD lines, (8) Grow best-predicted Syn-0 from best SSD lines, (9) Grow and test Syn-1, (10) Identify and multiply best synthetic cultivars. Star symbol indicates that information stems from field trials, seed from maintenance of SSD lines is taken for new crosses, for polycross and for Syn-0, respectively.

The principal methods used in the development of virtually all modern faba bean cultivars include mass selection, sophisticated methods of recurrent selection, and conventional methods of cultivar development such as phenotype-based pedigree selection, single-seed descent line breeding, or development of synthetic cultivars (Figure 1). For these methods, parental lines are chosen on the basis of their pedigree and their phenotypic attributes.

Faba bean is a partially allogamous species, and in principle, is amenable to line breeding, population breeding, and hybrid breeding. The degree of outcrossing of faba bean varies widely among genotypes (10–70%) and is highly affected by the environment (Link, 1990; Ederer and Link, 1993; Suso et al., 1999, 2001; Gasim et al., 2004), including the degree of heat stress (Bishop et al., 2016b), which is one of the major challenges of breeding the crop. Outcrossing is a poorly heritable character and is markedly influenced by the inbreeding coefficient of a genotype (Link, 1990; Brünjes and Link, 2021) and by the type of insect pollinators. Moreover, faba bean genotypes markedly differ in their ability to spontaneously self-fertilize, spontaneous meaning without access of their pollinators, which are primarily honeybees (*Apis mellifera*) and bumblebees (*Bombus hortorum* and other *Bombus* spp.) (Link, 1990; Torres et al., 1993; Bishop et al., 2020). Thus, a calamity occurs if breeders multiply pure lines under insect-proof enclosures. These genotypes fully depend upon insect pollinators and do not set seed in their absence without mechanical tripping of flowers. Auto fertility is defined as the ability of a plant to self-fertilize and hence set seed without being tripped (Drayner, 1956; Torres et al., 1993). Genetic variability for auto fertility has been reported in faba bean, and higher levels of auto fertility were reported in F1 hybrids than in inbred lines (Link, 1990; Bishop et al., 2020). Australian bred cultivars are highly auto fertile as their original genetic resources trace back to ICARDA germplasm that were mostly auto fertile. Furthermore, they are initially grown and selected under insect-proof enclosures. Depending on the level of outcrossing, faba bean breeding may be performed under conditions of controlled selfing in insect-proof cages or, with less control and in case of little outcrossing, based on developing lines under open field conditions (Gharzeddin et al., 2019).

Hybrid vigor (heterosis) is pronounced in faba bean. Heterozygous F1-hybrids exceed the yield of their homozygous parents by 40 to 70% (Zeid et al., 2004; Dieckmann and Link, 2010). Early approaches to hybrid breeding using cytoplasmic male sterility (CMS) system in faba bean trace back to David Bond in Cambridge, UK in 1957 and Pierre Berthelem in Rennes, France in 1967 (Pfeiffer et al., 1993; Link et al., 1997). To date, hybrid breeding is not established in this crop due to instability of the available CMS systems. An alternative to hybrid breeding is developing synthetic cultivars (i.e., a population breeding method). Breeding line cultivars and synthetic cultivars (or composite cultivars) are widely used products for faba bean improvement.

### Hybrid Breeding

There are three major obstacles to faba bean hybrid breeding. First, an operational CMS system must be available. The available CMS systems in faba bean have shown an insufficient level of male sterility. The pollen sterility is not very “deep” and not reliable, and frequent, spontaneous reversion to fertility occur (Link et al., 1997; Maalouf et al., 2019). The second obstacle is the availability of appropriate pollinators. Pollinators often “steal” nectar, removing it without placing pollen on the stigma of the flowers (Marzinzig et al., 2018). Pollinator insects, if gathering pollen, learn to avoid the pollen-free mother plants, thus limiting seed set in hybrid seed production (Bond, 1989a,b; Duc, 1997; Marzinzig et al., 2018; Brunet et al., 2019). Lastly, the low propagation coefficient of faba bean (Link and Stützel, 1995) requires three or more generations of seed increase from the manually tended, single row level of propagation to reach the seed quantity required for certified seed production. Thus, all operational steps and tools for hybrid seed production, including the reliability of the CMS system, must be flawlessly conducted to be successful.

Given the lack of CMS and gametocides, breeders have employed monogenic traits to support or realize hybrid seed production. These traits include recessive and dominant nuclear-genetic pollen sterility (Duc et al., 1985), testa color, hilum color, cotyledon color, flower color, and other plant morphological characteristics as markers. In addition, even seed size was used, although this is a multigenic trait (Link, 2009). Breeders have shown high levels of creativity over the decades for faba bean hybrid breeding but a breakthrough has yet to occur (Bond et al., 1966; Berthelem and Le Guen, 1967, 1974; Berthelem, 1970; Bond, 1989a,b; Link et al., 1994; Link, 1998; Duc and Stoddard, 2018).

### Line Breeding

Cultivars released via line breeding undergo strict selection intensity. One (the best) single line is developed into a cultivar, compared to two lines required to produce a hybrid, or more than three lines to develop a synthetic cultivar. Line cultivars can be developed faster than synthetic cultivars, since the latter need lines as components. Line cultivars are more widely accepted based on rules of distinctiveness, uniformity, and stability than synthetic cultivars. The single seed descent (SSD) procedure as proposed in Figure 1 may be substituted by using F_2_-derived lines (i.e., partial bulks) at F_3_ or advanced generations to test, select and hence release new cultivars, however, such cultivars can be relatively heterogeneous. This approach is focused on short breeding cycles and relies on high seed multiplication rates. Oftentimes, F_5_-derived or F_6_-derived lines are chosen as the basis for a new line cultivar and its maintenance breeding. Whichever approach is used for making lines, there is a need to multiply them under controlled self-fertilization or isolation: whether it is to initiate the multiplication as a line cultivar or to enter into the polycross or into a Syn-0 (Figure 1). The observation trial may be, focusing on synthetic cultivar breeding (see below), arranged as a soft version of a polycross (Ederer and Link, 1992a,b), with several rather than one plant per label and hence a reduced number of replicates; or, as top cross, with few replicates, and with half or less of the area devoted to a joint, constant pollinator genotype.

The low propagation coefficient of faba bean limits the breeding and especially the yield testing, compared to other crops. For example, one faba bean plant produces just enough seed for planting a 1 m^2^ of a field plot (about 20–40 seeds). Hence, one individual plant of an SSD pipeline, after controlled self-fertilization, gives just enough seed for a small observation trial; harvesting that plot would give enough seed for a test in about 20 to 40 m^2^ plot. This could be a trial in one location with two replicates; or two locations with one replicate including some checks in an augmented *p*-rep design (Moehring et al., 2013). A further point in conventional faba bean breeding is to optimize choice of parents. Focusing on line cultivars, basically half of the genetic variance of the breeding germplasm resides between the lines that result from the crosses that—potentially—can be made. The other half of the genetic variance lingers between these crosses, highlighting the importance of choice of parents (Falconer and Mackay, 1996) and therefore, it is of high importance to eliminate entire crosses that promise poorer performing lines than other crosses before investing too much in testing their offspring. Even if several crosses could be rejected as late as the observation trials in step 3 (Figure 1), then more plots of the more promising crosses could be tested in step 4. If, instead of SSD as in Figure 1, a pedigree-type of selection is installed, then as early as in generation F3 and F4, an observation trial or yield trial would allow to judge the yield potential of the entire cross (i.e., the entire family) and, as described above, eliminate inferior crosses (families). A corresponding marker investment should be made to select between crosses, before making them and after having made them.

### Synthetic Breeding

In synthetic breeding, usually four or more founder lines serve as components of a cultivar. The cultivar can, hence, be “re-synthesized” from these lines at any time. Selecting these inbred lines based on their *per se* performance (Figure 1, step 3) is probably suboptimal because the synthetic population will not be as highly inbred as its components. The purpose of choosing synthetic breeding instead of line breeding is indeed to exploit heterosis as much as possible for agronomic performance, i.e., to decrease inbreeding (Ghaouti et al., 2008; Ghaouti and Link, 2009). Inbred lines as synthetic components should hence be selected mainly for their so-called varietal ability. This parameter is associated with general combining ability and breeding value (Cockerham and Weir, 1984; Ederer and Link, 1993). The varietal ability of a candidate line is approximately realized by the performance of its polycross-progeny or top-cross progeny (Maalouf et al., 1999). To assess, in addition to this, the line's *per se* performance, and even more to assess their degree of cross-fertilization and their paternal success, this all might allow to better predict the performance of the potential synthetics. Yet these additional parameters might not be worthwhile; assessing them might not be the optimum allocation of breeders' budget (Edere and Link, 1992; Ederer and Link, 1993; Brünjes and Link, 2021). *Per se* yield of lines and yield of their polycross progenies were correlated and had a genetic correlation coefficient (r_G_) = 0.51 (Fleck and Ruckenbauer, 1989). Ghaouti et al. (2008) reported a slightly higher correlation (r_G_ = 0.63) between inbred lines and their polycross progenies for yield performance. Due to this, and because only slight corrections can be realized if *per se* performance is available in addition to polycross-progeny data, yield-testing of the lines may not be adequate (Ederer and Link, 1993). Yield testing of the lines themselves is expensive, since seed has to be multiplied under conditions of controlled selfing, whereas seed production for yield testing of progenies (from polycross or from top cross) is cheaper.

The terms polycross and top cross need not be taken literally. First, both options may serve a very similar purpose. A top cross may be easier to be conducted than a polycross. The pollinator (tester) of a top cross might be sown in strips, such as with 50% or less of the field area, the candidates would mainly cross-pollinate with the common pollinator, according to their individual extent of outcrossing.

The discrepancy between the line's *per se* performance and the performance of their open pollinated offspring (polycross-progenies; top cross-progenies) is caused by several reasons (beyond the deviations of randomness in the outcrossing that may come from compromises in the experimental and field lay-out; from pollinator behavior; from differences in paternal outcrossing success of the lines; Brünjes and Link, 2021). The major source of this discrepancy is General combining ability, which is a component of the polycross/top cross progenies' performance, and this is line-specific. The same applies for the degree of self-fertilization of the candidate lines and the paternal outcrossing success. Consequently, the level of inbreeding and actual composition of the progenies is line-specific. Yet, and moreover, the seed for inbred lines is produced in cages, seed for open-pollinated progenies is produced in open field, hence in different environmental conditions. Thus, when comparing performance of lines with that of their open-field derived progenies, seed size and seed quality and thus the resulting crop stands are expected to differ for non-genetic reasons (Bond and Pope, 1984).

Ederer and Link (1992a,b) simulated the relative importance of *per se* yield of lines, of general combining ability of lines, and of the degree of cross-fertilization of lines for the performance of synthetics (Syn-1, Syn-2 and Syn-3). The coefficient of determination (*R*^2^) for *per se* yield as an independent variable varied from 0.41 to 0.64 (depending on the number of components and Syn generation). The *R*^2^ of general combining ability as an independent variable ranged accordingly from 0.63 to 0.75. For the degree of cross-fertilization, the simulation *R*^2^ was between 0.02 and 0.17, with the higher values for Syn-1 and for four instead of eight components.

Link et al. (1991) analyzed phenotypic data from 36 spring faba bean lines along with nine synthetic populations (each derived from a quartet with four founders) across 12 environments in south Germany from 1986 to 1990. Their results showed that for days to anthesis, days to maturity, thousand seed weight, plant height and lodging, the *R*^2^ of the averages of nine line quartets and the results of their nine synthetic populations was on average 0.84 (half of the associations were *R*^2^ > 0.90). The *R*^2^ for the association between the nine line quartets with yield of their synthetic populations was only 0.90 for Syn-0, 0.62 for Syn-1 and 0.48 for Syn-2. This decline through the synthetic generations very often follows the increasing difference in heterogeneity and heterozygosity between the (quartets of the) homozygous lines and the successive synthetic generations.

These considerations support to the notion that additional assessment of the *per se* performance of lines and hence a focus on the difference between *per se* performance and polycross/top cross-progeny performance may not be worthwhile, but rather all budgets may be devoted to field-phenotype the open-field derived progenies of the candidate lines.

It is not self-evident whether several synthetic populations have to be developed and tested, to select between them; or whether only those synthetics should actually be constructed to be further submitted to the official trials. Selecting between lines first based on observation trials (Figure 1) and then based on data of their polycross/top cross-progenies, then selecting between predicted (but not yet realized) synthetic cultivars may help focus resources on only the very-best predicted synthetic population(s) (Ederer and Link, 1992a,b, 1993; Schill et al., 1992).

### Mutagenesis in Faba Bean

Novel mutations on certain faba bean genes will enrich its diversity for breeding. Such mutants may be induced by chemical mutagenesis or irradiation. Physical (e.g., gamma-ray) and chemical (e.g., ethyl methane sulphonate-EMS) mutagens have demonstrated their usefulness in faba bean mutation induction (Abdalla, 1982; Duc, 1995; Khursheed et al., 2018, 2019; Nurmansyah et al., 2019, 2020). Ion beam irradiation has also been reported as an effective and unique technique for inducing mutations in faba bean (Khazaei et al., 2018b). The first mutagenesis on faba bean was reported by Sjödin (1971) with the description of determinate mutants, *ti1* and *ti2* which significantly reduced the number of flowering nodes giving the plant type as “topless.” Later, van Norel and Hoogendoorn (1989) reported a dwarf mutant, *dw1*, which reduced the internode length by almost 50%. Ramsay et al. (1991) reported a gene for reduced vicine-convicine content, *vcr*, and Duc (1995) found five nodulation mutants through EMS mutagen where one of them was a super modulating mutant (*f32*) giving 3-5 times more nodules than the normal type. Bhatia et al. (2001) has given a detailed description of 265 varieties developed through induced mutation on grain legumes including 13 in faba bean genotypes. A number of imazapyr resistance mutations through EMS were identified in South Australia (Mao et al., 2019) that resulted in the development of the first imidazolinone herbicide resistant variety, PBA Bendoc in Australia. Recently morphological diversity of faba bean mutant populations was explored (Nurmansyah et al., 2019, 2020). Mutation on grain legumes including faba bean was reviewed by Huyghe (1998). A number of mutation breeding projects across the world have proven the efficiency of mutagenesis in grain legumes to broadening the genetic variation (e.g., Micke, 1984; Tadege et al., 2009; Horn et al., 2016; Raina and Khan, 2020).

Seeking mutants that increase resistance against the root parasitic weeds broomrape and stem parasitic dodders, both being increasingly a problem in the Mediterranean basin, will promote faba bean production in this region; resistance of faba bean against herbicides that control these weeds would especially be useful and is a sought-for trait (Rubiales and Fernández-Aparicio, 2012; Fernández-Aparicio et al., 2016; Rubiales et al., 2016; Mejri et al., 2018). Similarly, mutants that decrease pod wall thickness will increase grain yield. Inbred lines harboring new diversity such as increased resistance or improved quality, accruing from recombination-fueled transgression or from mutagenesis (Bhatia et al., 2001) may be used at step (1) in Figure 1. Currently 20 faba bean mutant varieties have been developed from various countries which are listed at IAEA (International Atomic Energy Agency, 2021) mutant variety database (https://bit.ly/3yVEReM) that might be used in breeding programs. Mutagenesis combined with biotechnology tools may accelerate releasing novel faba bean germplasm and improved cultivars.

### Speed Breeding

Speed breeding to shorten breeding cycles is a must have tool in any breeding programs for the purpose of increasing genetic gains per year. Significant progress has been made toward shortening the reproductive cycle and hence the overall process of cultivar development (see below) as a tool for accelerating the breeding of pulse crops (*reviewed by* Cazzola et al., 2021). For example, Samineni et al. (2020) reported that up to seven generations/year can be obtained in chickpea without applications of chemical treatment. Recently an *in vivo* speed breeding protocol, for the first time, for faba bean has been reported by Mobini et al. (2020). This can be a valuable tool for developing diverse germplasm and improved cultivars in a relatively short time span. They reported that application of cytokinin or cold treatment could increase pollen viability and seed setting, thus consequently decreasing the length of the breeding cycle in faba bean by 22 days from seed to seed. Additional reduction in faba bean generation time as part of a reliable speed breeding protocol may be obtained by implementing accelerated faba bean development to the flowering stage by regulating light and temperature. Recently, this has been achieved in Australia where five generations per year can be obtained (Janine Croser, 2021, pers. comm). Speed breeding can accelerate development of faba bean lines and may be integrated with other cutting-edge breeding tools such as marker assisted breeding and genomic selection based on estimated breeding values (Bhatta et al., 2021). However, due to the tiny amount of seed produced on a typical speed-bred plant and the expense of maintaining controlled environments, speed breeding protocols are only suited to the primary cross/backcross and inbreeding generations of a breeding scheme, pending further research to extend the applications of speed breeding. All steps toward phenotyping of agronomic performance rely on considerable seed increase, probably in open pollination conditions. Phenotypic evaluations must eventually be carried out under “natural” conditions.

## Marker-Assisted Selection and DNA Marker Availability

Marker-assisted selection is an indirect selection process using genetic markers that are tightly linked to traits of interest but are difficult or costly to phenotype. Selection on the basis of a DNA markers can be done for a fixed and predictable cost, whereas phenotypic selection may require either a dedicated, expensive or destructive screen, and deliver results that are still in part depending on the interactions of the genotypes with the actual conditions of such a test. A good example that illustrates how DNA markers could be implemented in faba bean breeding is the case of vicine and convicine (v-c), the main anti-nutritional factors limiting faba bean seed usage. The v-c causes favism in pre-disposed humans and lowers feed conversion efficiency in monogastric animals and therefore removal of v-c by genetic means is unequivocally desirable. Some decades ago, a natural low v-c variant was identified, and shown to be controlled by a single gene (Duc et al., 1989). This gene was mapped to the tip of faba bean chromosome 1 and a diagnostic SNP assay for use in selection was developed (Khazaei et al., 2015, 2017). It is now known that low v-c faba bean cultivars carrying the *vc*^−^ gene are safe for favism sufferers. All elements are in place for straightforward foreground selection of low v-c genotypes, thereby bypassing the need for expensive, time-consuming and destructive biochemical analysis of seeds (Khazaei et al., 2019a; Tacke et al., 2021). The vc^−^ marker and another marker for a herbicide (imidazolinone) tolerance (Mao et al., 2019) are being used routinely in faba bean breeding programs in Australia. Very recently, the biosynthetic pathway of v-c was uncovered (Björnsdotter et al., 2021). Similarly, robust DNA markers for low seed coat tannins *zt1* (Webb et al., 2016; Gutierrez and Torres, 2019) and *zt2* (Gutierrez et al., 2020; Zanotto et al., 2020), growth habit (*Vf_TFL1*, Avila et al., 2006, 2007) and rust resistance genes (*Uvf1* and *Uvf2*, Ijaz et al., 2021b) have been developed. Furthermore, closely linked DNA markers have been reported for host plant resistance to ascochyta blight (Avila et al., 2004; Kaur et al., 2014; Atienza et al., 2016; Sudheesh et al., 2019; Faridi et al., 2021), however, their verification and availability in breeder friendly format is yet to come. Detailed information on available QTLs and DNA markers for adapting to biotic and abiotic stresses in faba bean was recently reviewed by Khazaei et al. (2021). Faba bean breeding efforts would benefit greatly from development of effective DNA markers for improving resistance to chocolate spot.

## Genomic Selection

Low-cost genome-wide genotyping and high-throughput phenotyping platforms open up the further possibility of speeding up selection for complex traits. Genomic selection by definition focuses on multigenic traits and is rather a black-box approach than the attempt to analyze and follow-up the genetic variation locus-by-locus and candidate-gene-wise. Genomic selection has been shown to be a useful tool in recurrent selection within base populations of allogamous crop species that are constructed as a synthetic population (Müller, 2017). Accuracy values for grain yield are typically lower than heritability values (or their square roots) from a series of yield trials. Yet, genomic predictions are conducted for individuals. Breeding values for grain yield or for agronomic performance, genomically estimated for single plants, is tremendously more precise than heritability of such single plants' performance. This allows genomic selection to be implemented based on single plants, and it especially allows markedly shortening of breeding cycles; the time-consuming phase of developing and phenotyping inbred lines can be eliminated from the selection and recombination phase of breeding and can be shifted to the cultivar-making phase. Genomic selection has much to offer faba bean breeding, provided that a highly cost-effective genome-wide genotyping platform becomes available.

Genomic selection may accelerate faba bean breeding by reaching a cross-to-cross cycle length of 6 months to 1 year, thereby increasing genetic gain per unit time. One possible implementation of genomic selection that would work well in faba bean is the two-part breeding strategy proposed by Gaynor et al. (2017) with application of genomic selection mainly in the first (population improvement) component of the scheme. The breeding population, i.e., the breeding germplasm, has to be genetically elite and diverse. Due to the partial allogamy, the individuals are more or less heterozygous. This population is genotyped, with a very high number of (representative) individuals. Genotyping is conducted on seeds instead of plants (chipped seed, Mills et al., 2020) and genotypic data are used to predict line *per se* performance or varietal ability (see above). Selection is applied accordingly, between chipped seeds. Then only the selected chipped seed is sown as a new generation of the breeding population, and is directly allowed to propagate further according to its natural, partially allogamous mode, under open pollination, without any intermittent phase of inbreeding or multiplication or phenotyping. A large, representative sample of offspring (seed) from this population is again genotyped, genomic estimated values are predicted and selection is again applied; and so forth. It is possible in this way, even without the benefit of speed breeding, to achieve two cycles per year. This rapid turnover may be termed “recurrent genomic selection.” The process of creating inbred lines from the most promising, selected individuals is organized as a separate pipeline, which may be termed “cultivar development” or “product development” (Gaynor et al., 2017). Phenotypic data to steer and adjust the training of the algorithm for the genomic prediction is gathered during cultivar development (either for line or synthetic cultivars).

**Figure 2:**
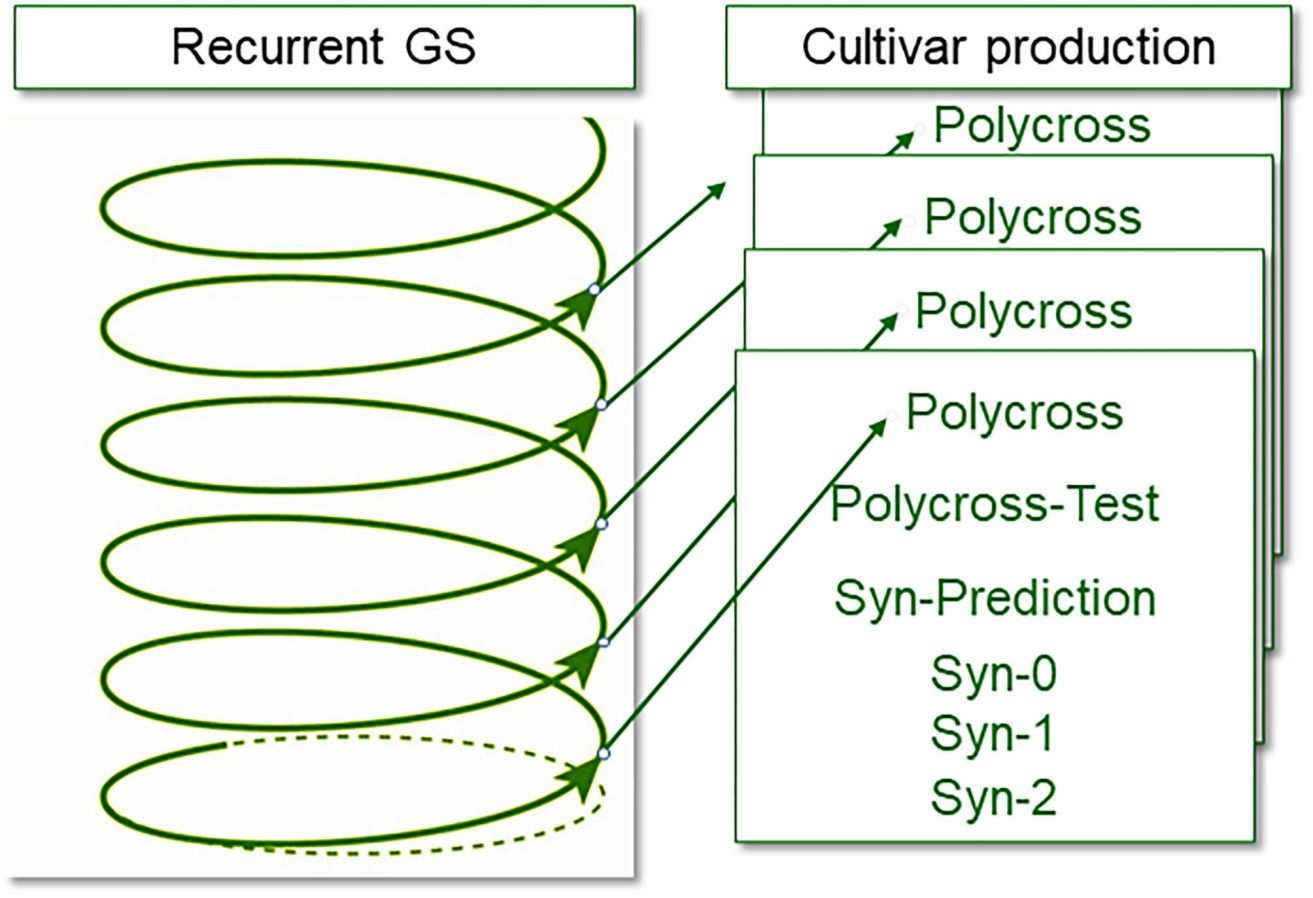
Separation of recurrent genomic selection and recombination (based on single plants of a breeding population) from the actual cultivar development. Selection in the cultivar production is partly genomic data and partly phenotypic data (adapted from Gaynor et al., 2017).

The partially allogamous nature of faba bean offers the recombination that is necessary for the fast-cycling recurrent selection and the self-fertilization that is required for the production of inbred material for cultivar development synchronously and at zero cost (Table 1). To enter the cultivar development with inbred lines, the only task is—via genotyping—to identify the inbreeding levels of the individuals of the breeding population (the population under recurrent genomic selection, Figure 2, Table 1) and find the more inbred ones. With genomic selection, the individuals in this fast-cycling population require genotyping anyway, and the inbred levels of the individuals accrue as a side product. The inbred individuals that enter the cultivar development pipeline transfer the level of genetic improvement that is realized in the recurrent population improvement. During cultivar development, genomic selection and phenotypic selection will both be applied. Selection will be based on an index value from phenotypic data and genomic estimated data. The scheme would be very similar to conventional line breeding and synthetic breeding. The maintenance breeding would immediately start as an inbred individual enters the cultivar development.

**Table 1:** Theoretical composition of a partial allogamous population Cockerham and Weir, 1984, with 20% cross-fertilization of non-inbred individuals and 50% cross-fertilization of homozygous individuals (linear relationship between cross-fertilization and inbreeding; Link, 1995; Brünjes and Link, 2021).

**Parameter**	**Hybrids**	**Intermediate generations**			**Highly inbred**	
	**F1**	**F2**	**F3**	**F4**	**F5**	**F5>**
Inbreeding coefficient	0.000	0.500	0.750	0.875	0.938	>0.968
Frequency in population	0.339	0.271	0.176	0.101	0.055	0.057

## Phenotypic Recurrent Selection of Populations

The proposed two-part genomic selection strategy adapted for faba bean as outlined above has not yet been implemented in practice. Utilizing currently available genotyping platforms (individual KASP assays or high density Axiom array), the cost of eliminating a genetically inferior individual plant by genome-wide genotyping is still beyond the means of most private or public breeding programs. However, what if phenotypic selection were performed on the basis of individual plant seed yield? This approach has been avoided in the past as an individual plants agronomic performance e.g., for yield, is considered to have low heritability, and in the context of heterogeneous crop stands, to be confounded by selection on the probably correlated trait of competitive ability, requiring elaborate honeycomb designs to circumvent (reviewed in Fasoula and Tokatlidis, 2012). However, selection on a single plant basis can be conducted at a very low cost per genotype evaluated, since only the top 5-10% of the population would need to be individually threshed and weighed, the rest being eliminated by eye or by recording the weight of the unthreshed plant requiring just seconds of evaluation time, and with none of the time required to inbreed and bulk. In fact, rapid recurrent selection based on phenotype has been empirically tested recently, with promising results. Tagkouli (2020) set up a faba bean polycross population with 22 founding cultivars and lines, using captive bumblebee colonies to maintain high outcrossing rates in each generation, and selecting individual plants with the highest seed yield in each of three successive field seasons in the UK (Reading) and two seasons in Ireland (Carlow). The respective populations selected were reciprocally tested across sites and their yield performance compared to elite spring varieties. Although no differences in these trials passed the thresholds for statistically significant differences, the thrice-selected Reading selected population outperformed all earlier selection generations as well as all elite checks in the Reading trial whereas this result was partly reversed in the reciprocal trial in Ireland. Detailed study of the Reading population performance in Reading showed this genetic gain was associated with a marked increase in hundred seed weight, faster emergence and canopy closure and a marked increase to very high levels of heterozygosity as judged by genome-wide genotyping of the selected individuals at each selective generation. Furthermore, signatures of purifying selection were detected at a number of specific loci, suggesting that the rapid turnover of highly recombined, highly heterozygous populations enabled rapid adaptation to environment through enrichment of alleles that confer selective advantage concurrently with optimizing levels of hybrid vigor in the population.

This observation that hybrid vigor is the major factor determining population performance was also noted by Ghaouti and Link (2009), who showed that average polycross progeny yield was greater than either the average of the inbred polycross parents (*n* = 18), or indeed the predicted yield of the best possible four-component synthetic that could be drawn from the 18 inbred lines, so this outcome of rapid recurrent selection is not surprising. In fact, where the natural environment exercises sufficient selection, mass selection alone can result in rapid adaptation of populations, as shown recently by Landry et al. (2017) for the case of development of winter-hardy faba bean populations for the US Pacific North-West.

The question that remains to be investigated is the relative efficiency and cost-benefit of genomic selection over phenotypic selection.

## Genetic Gain

In the absence of a reference genome assembly for faba bean, high-density genetic maps and high-throughput approaches such as transcriptome analysis have been used for enrichment of genomic resources (Khazaei et al., 2021). At the writing of this article various cooperative reference genome assembly efforts are concurrently underway. The development of a reference genome will provide a foundational resource for faba bean research to accelerate genetic gain. Speeding up the translation of genomic knowledge into plant breeding would lead to accelerated genetic gain. By integrating genomic knowledge with other breeding tools and platforms will enable to accelerate the breeding process for enhancing genetic gain for faba bean. Translational research will require coordination and understanding between laboratory-based and field-based scientists that can work as a bridge between fundamental research and practical plant breeding.

The major pulse crops have not experienced expanded production and consumption at a uniform rate over the past 50 years. Lentil production has expanded at more than 10% annually, chickpea and dry bean at 2 to 3%, and pea at <1%; while faba bean production declined sharply until 1992 and it was practically recuperated in 2019 due to the productivity increases from <1 to 2.1 t ha^−1^ while the human population has increased at 2.4% annually (Khazaei et al., 2019b; FAOSTAT, 2021). Now that environmental and nutritional concerns combined with demand for plant protein have gained considerable momentum on a global scale, the improvement and expansion of yield potential, sustainable production stability and food industry quality of faba bean are more important than ever before.

## The Future of Faba Bean Breeding

Faba bean is a biologically and genetically unique crop that has much to offer both in terms of its nutritional density and agro ecological role in reducing fertilizer requirements and supporting pollinator populations. Agricultural policy makers and funders should be aware that there are many enticing opportunities to accelerate genetic gain in faba bean.

The conquering of the bloated 13 Gb diploid genome at last (fabagenome.dk) affords a timely opportunity to develop cheap genotype-by-sequencing approaches already common in other species. This is because low cost, low complexity skim sequencing requires a robust and complete reference genome assembly to map short reads to, whereas until now, the only successful genotyping approaches have required targeting of each individual locus. Cheap genotyping-by-sequencing (GBS) should usher in a new era of genomic selection; however, hurdles are not just on the genotyping side. Modeling that takes the mixed mating system into account and develops population-and site-specific estimates of outcrossing rate, seed multiplication rate and trait heritability's is needed to ensure that genomic selection gives significant and cost-effective increases in the rate of genetic gain.

Burgeoning genome knowledge can be exploited in other arenas. A facile, high efficiency transformation system would enable gene editing of a growing list of genes whose function is relatively well-understood in related legumes at least, if not in faba bean. Clearly, there remains uncertainty over the timescale over which gene edited crops could become widely accepted, but we should take the long-term view and fund the underpinning work necessary to bring forward case studies by which the cost-benefit analysis can be done for specific applications. Meanwhile, classical mutagenesis, newly invigorated by cheap re-sequencing technology (Wang H. et al., 2012), may soon allow *in silico* mutagenesis to become a reality.

The ability to obtain an allelic series of mutants in specific target genes such as CENH3 either via gene editing or mutagenesis (Jacquier et al., 2020) should enable the production of doubled haploid inducer lines, which would immediately impact the rate of genetic gain, possibly even more than “speed breeding,” while a cytoplasmic male sterility system, naturally occurring or transgenic, would enable the topic of hybrid breeding to be revisited. Up to this point, our pre-breeding research wish list could have applied to virtually any crop. However, faba beans' unique characteristics mean that we cannot simply adopt a generic blueprint for high-tech breeding technologies (i.e., from maize) without regard for the biological specificities of the crop.

The mutualistic relationship of faba bean with its insect pollinators needs more detailed study, both to understand better how natural pollinator populations can be nurtured to enhance long-term yield stability in the face of climate change (Bishop et al., 2016a), and to use pollinator-assisted outcrossing more effectively as a breeding and seed propagation tool (Tagkouli, 2020).

A related, longer-term objective relates to understanding genetic control of pollinator attraction and pollinator function traits on which the efficiency of pollinator services depend (Suso et al., 2016; Bailes and Glover, 2018; Bailes et al., 2018). Such an understanding would underpin the opposite but non-mutually exclusive goals of breeding for optimal interaction with a specific pollinator or breeding for complete pollinator independence (i.e., conversion of faba bean to a fully autogamous mating system).

Finally, the genetic control of the symbiotic relationship with rhizobia to develop nitrogen-fixing nodules also merits further investigation as it is clear there is unexplored variation in the rhizobial population for nodulation efficiency and nitrogen fixation rate and an unexplored variation in the faba bean population for strain selectivity, all of which needs to be understood in the context of highly variable soil physical and chemical properties and the resident soil microbiome.

## Conclusions and Future Perspectives

In spite of the fact that faba bean has demonstrably the highest nitrogen fixation capability among grain legumes, high protein content increasing interest in faba bean *per se*, its production has not been expanded. Now that the environmental consequences of crop production and food systems are under intense scrutiny, faba bean is set to become an important source of plant protein. Increasing faba bean production will have impact on cropping systems that include other pulse crops. For example, faba bean can be used to extend temperate crop rotations that include field pea and lentil which are increasingly at high risk of yield loss due root rot caused by *Aphanomyces euteiches* (Karppinen et al., 2020). Demand for plant-based protein for food systems is rapidly expanding, and faba bean is an excellent source of protein flour because of its bland flavor and light color. The seed size of faba bean can be reduced without affecting yield potential, thereby lowering the costs of production. Seed shape can be changed from flat to round, making it more amenable to mechanical seeding systems that are widely used for soybean, allowing the seed distribution uniformity that will maximize yield, reduce seeding costs, and potentially reduce the spread of diseases that affect the canopy. Reducing seed size has the potential to reduce the overall costs of breeding per unit of genetic gain, and also reduce the overall costs of both breeding and commercial production for breeding programs targeting the protein extraction industry. All of these goals can be achieved more quickly by a coordinated breeding approach that focuses on maximizing genetic gain using a combination of newly available genetic technologies and focusing on breeding objectives that go beyond the traditional market place for faba bean.

## Data Availability Statement

The original contributions presented in the study are included in the article/Supplementary Material, further inquiries can be directed to the corresponding author.

## Author Contributions

All authors listed have made a substantial, direct, and intellectual contribution to the work, and approved it for publication.

## Funding

KA research was supported by the Australian Grains Research and Development Corporation, UA00163. WL research was supported by the EU-project SusCrop Call 1 (ProFaba) via the German BMBF (Grant Number 031B0805A). DO'S research was funded by the Defra Pulse Crop Improvement Network (CH0110) and BBSRC grant BB/P023509/1. FM research was funded by the bilateral Grant of Egypt bilateral program 2019-2020 (Grant number 200193). AV and HK research was supported by the ADF (Agriculture Development Fund–Government of Saskatchewan, Canada; Grant Number: 20150285), the WGRF (Western Grains Research Foundation, Canada; Grant Number: VarD1609), the SPG (Saskatchewan Pulse Growers, Canada; Grant Number: BRE1714), and NORFAB (Innovation Fund Denmark grant number 5158-00004B).

## Conflict of Interest

The authors declare that the research was conducted in the absence of any commercial or financial relationships that could be construed as a potential conflict of interest.

## Publisher's Note

All claims expressed in this article are solely those of the authors and do not necessarily represent those of their affiliated organizations, or those of the publisher, the editors and the reviewers. Any product that may be evaluated in this article, or claim that may be made by its manufacturer, is not guaranteed or endorsed by the publisher.

## References

[B1] Abdalla M. M. F. (1982). Mutation breeding in faba beans, in Faba Bean Improvement. World Crops: Production, Utilization, and Description, Vol. 6, eds Hawtin G. Webb C. (Dordrecht: Springer), 83-90. 10.1007/978-94-009-7499-9_8

[B2] Abdelmula A. A. Link W. von Kittltz E. Stelling D. (1999). Heterosis and inheritance of drought tolerance in faba bean, *Vicia faba* L. Plant Breed. 118, 485–490. 10.1046/j.1439-0523.1999.00411.x

[B3] Abebe T. Meles K. Nega Y. Beyene H. Kebede A. (2013). Interaction between broomrape (*Orobanche crenata*) and resistance faba bean genotypes (*Vicia faba* L.) in Tigray region of Ethiopia. Can. J. Plant Protect. 1, 104–109.

[B4] Adhikari K. N. Zhang P. Sadeque A. Hoxha S. Trethowan R. (2016). Single independent genes confer resistance to faba bean rust (*Uromyces viciae-fabae*) in the current Australian cultivar Doza and a central European line Ac1655. Crop Pasture Sci. 67, 649–654. 10.1071/CP15333

[B5] Alharbi N. (2018). Factors of yield determination on faba bean (Vicia faba L.) as influenced by varying sowing times (PhD thesis). School of Life and Environmental Sciences, The University of Sydney, Sydney, Australia.

[B6] Alharbi N. H. Adhikari K. N. (2020). Factors of yield determination in faba bean (*Vicia faba*). Crop Pasture Sci. 71, 305–321. 10.1071/CP19103

[B7] Amede T. Kittlitz E. V. Schubert S. (1999). Differential drought responses of faba bean (*Vicia faba* L.) inbred lines. J. Agron. Crop Sci. 183, 35–45. 10.1046/j.1439-037x.1999.00310.x

[B8] Arbaoui M. Balko C. Link W. (2008). Study of faba bean (*Vicia faba* L.) winter-hardiness and development of screening methods. Field Crops Res. 106, 60–67. 10.1016/j.fcr.2007.10.015

[B9] Asif M. A. Paull J. G. (2021). An approach to detecting quantitative trait loci and candidate genes associated with salinity tolerance in faba bean (*Vicia faba*). Plant Breed. 140, 643–653. 10.1111/pbr.12934

[B10] Atienza S. G. Palomino C. Gutiérrez N. Alfaro C. M. Rubiales D. Torres A. M. . (2016). QTLs for ascochyta blight resistance in faba bean (*Vicia faba* L.): validation in field and controlled conditions. Crop Pasture Sci. 67, 216–224. 10.1071/CP15227

[B11] Avila C. M. Atienza S. G. Moreno M. T. Torres A. M. (2007). Development of a new diagnostic marker for growth habit selection in faba bean (*Vicia faba* L.) breeding. Theor. Appl. Genet. 115:1075. 10.1007/s00122-007-0633-y 17828523

[B12] Avila C. M. Nadal S. Moreno M. T. Torres A. M. (2006). Development of a simple PCR-based marker for the determination of growth habit in *Vicia faba* L. using a candidate gene approach. Mol. Breed. 17, 185–190. 10.1007/s11032-005-4075-4

[B13] Avila C. M. Satovic Z. Sillero J. C. Rubiales D. Moreno M. T. Torres A. M. (2004). Isolate and organ-specific QTLs for ascochyta blight resistance in faba bean. Theor. Appl. Genet. 108, 1071–1078. 10.1007/s00122-003-1514-7 15067393

[B14] Avila C. M. Sillero J. C. Rubiales D. Moreno M. T. Torres A. M. (2003). Identification of RAPD markers linked to the *Uvf-1* gene conferring hypersensitive resistance against rust (*Uromyces viciae-fabae*) in *Vicia faba* L. Theor. Appl. Genet. 107, 353–358. 10.1007/s00122-003-1254-8 12698251

[B15] Bailes E. J. Glover B. J. (2018). Intraspecific variation in the petal epidermal cell morphology of *Vicia faba* L. (Fabaceae). Flora 244–245, 29–36. 10.1016/j.flora.2018.06.005 30008511 PMC6039855

[B16] Bailes E. J. Pattrick J. G. Glover B. J. (2018). An analysis of the energetic reward offered by field bean (*Vicia faba*) flowers: nectar, pollen, and operative force. Ecol. Evol. 8, 3161–3171. 10.1002/ece3.3851 29607015 PMC5869266

[B17] Belachew K. Y. Stoddard F. L. (2017). Screening of faba bean (*Vicia faba* L.) accessions to acidity and aluminium stresses. PeerJ 5:e2963. 10.7717/peerj.2963 28194315 PMC5301972

[B18] Berthelem P. (1970). Rapport d'activité 1967-1970. Rennes: INRA.

[B19] Berthelem P. Le Guen J. (1967). Rapport d'activité 1967. Rennes: INRA.

[B20] Berthelem P. Le Guen J. (1974). Rapport d'activité 1971-1974. INRA, Rennes.

[B21] Beyene A. T. Derera J. Sibiya J. (2018). Genetic variability of faba bean genotypes for chocolate spot (*Botrytis fabae*) resistance and yield. Euphytica 214:132. 10.1007/s10681-018-2210-7

[B22] Beyene A. T. John D. Sibiya J. Fikre A. (2016). Gene action determining grain yield and chocolate spot (*Botrytis fabae*) resistance in a faba bean. Euphytica 207, 293–304. 10.1007/s10681-015-1536-7

[B23] Bhatia C. R. Maluszynski M. Nichterlein K. van Zanten L. (2001). Grain Legume Cultivars Derived from Induced Mutations, and Mutations Affecting Nodulation. Vienna: IAEA. Available online at: https://www.osti.gov/etdeweb/biblio/20169549 (accessed September 1, 2021).

[B24] Bhatta M. Sandro P. Smith M. R. Delaney O. Voss-Fels K. P. Gutierrez L. . (2021). Need for speed: manipulating plant growth to accelerate breeding cycles. Curr. Opin. Plant Biol. 60:101986. 10.1016/j.pbi.2020.101986 33418268

[B25] Bhowmik P. Konkin D. Polowick P. Hodgins C. L. Subedi M. Xiang D. . (2021). CRISPR/Cas9 gene editing in legume crops: opportunities and challenges. Legum. Sci. 1–16. 10.1002/leg3.96

[B26] Birch A. N. E. Tithecott M. T. Bisby F. A. (1985). *Vicia johannis* and wild relatives of the faba bean: a taxonomic study. Econ. Bot. 39, 177–190. 10.1007/BF02907843

[B27] Bishop J. Garratt M. P. D. Breeze T. D. (2020). Yield benefits of additional pollination to faba bean vary with cultivar, scale, yield parameter and experimental method. Sci. Rep. 10:2102. 10.1038/s41598-020-58518-1 32034193 PMC7005869

[B28] Bishop J. Jones H. E. O'Sullivan D. M. Potts S. G. (2016b). Elevated temperature drives a shift from selfing to outcrossing in the insect-pollinated legume, faba bean (*Vicia faba*). J. Exp. Bot. 68, 2055–2063. 10.1093/jxb/erw430 27927999 PMC5429019

[B29] Bishop J. Potts S. G. Jones H. E. (2016a). Susceptibility of faba bean (*Vicia faba* L.) to heat stress during floral development and anthesis. J. Agron. Crop Sci. 202, 508–517. 10.1111/jac.12172 29353966 PMC5763371

[B30] Björnsdotter E. Nadzieja M. Chang W. Escobar-Herrera L. Mancinotti D. Angra D. . (2021). VC1 catalyzes a key step in the biosynthesis of vicine in faba bean. Nature Plants 7, 923–931. 10.1038/s41477-021-00950-w 34226693 PMC7611347

[B31] Bond D. A. (1989a). Prospects for commercialisation of F1 hybrid field beans *Vicia faba* L., Euphytica 41, 81–86. 10.1007/BF00022415

[B32] Bond D. A. (1989b). A short review of research on male sterility and prospects for F1 hybrid varieties in field beans (*Vicia faba* L.). Euphytica 41, 87–90. 10.1007/BF00022416

[B33] Bond D. A. Pope M. (1984). Effect of seed source on performance of faba bean varieties, in Vicia faba: Agronomy, Physiology and Breeding. World Crops: Production, Utilization, Description, *Vol. 10*, eds Hebblethwaite P. D. Dawkins T. C. K. Heath M. C. Lockwood G. (Dordrecht: Springer), 127–133. 10.1007/978-94-017-3647-3_13

[B34] Bond D. A. Fyfe J. L. Toynbee-Clarke G. (1966). Male sterility in field beans (*Vicia faba* L.). III. Male sterility with a cytoplasmic type of inheritance. J. Agric. Sci. 66, 369–377. 10.1017/S002185960006367X

[B35] Bond D. A. Jellis G. J. Rowland G. G. Le Guen J. Robertson L. D. Khalil S. A. . (1994). Present status and future strategy in breeding faba beans (*Vicia faba* L.) for resistance to biotic and abiotic stresses. Euphytica 73, 151–166. 10.1007/BF00027191

[B36] Bouhassan A. Sadiki M. Tivoli B. (2004). Evaluation of a collection of faba bean (*Vicia faba* L.) genotypes originating from the Maghreb for resistance to chocolate spot (*Botrytis fabae*) by assessment in the field and laboratory. Euphytica 135, 55–62. 10.1023/B:EUPH.0000009540.98531.4d

[B37] Brunet J. Zhao Y. Clayton M. K. (2019). Linking the foraging behavior of three bee species to pollen dispersal and gene flow. PLoS ONE 14:e0212561. 10.1371/journal.pone.0212561 30807591 PMC6391023

[B38] Brünjes L. Link W. (2021). Paternal outcrossing success differs among faba bean genotypes and impacts breeding of synthetic cultivars. Theor. Appl. Genet. 134, 2411–2427. 10.1007/s00122-021-03832-z 33961063 PMC8277637

[B39] Caracuta V. Weinstein-Evron M. Kaufman D. Yeshurun R. Silvent J. Boaretto E. (2016). 14,000-year-old seeds indicate the Levantine origin of the lost progenitor of faba bean. Sci. Rep. 6:37399. 10.1038/srep37399 27876767 PMC5120295

[B40] Cazzola F. Bermejo C. J. Gatti I. Cointry E. (2021). Speed breeding in pulses: an opportunity to improve the efficiency of breeding programs. Crop Pasture Sci. 72, 165–172. 10.1071/CP20462

[B41] Chen H. Richardson A. E. Rolfe B. G. (1993). Studies of the physiological and genetic basis of acid tolerance in *Rhizobium leguminosarum* biovar *trifolii*. Appl. Environ. Microbiol. 59:1798. 10.1128/aem.59.6.1798-1804.1993 16348956 PMC182164

[B42] Chooi W. Y. (1971). Variation in nuclear DNA content in the genus *Vicia*. Genetics 68, 195–211. 10.1093/genetics/68.2.195 17248534 PMC1212649

[B43] Cockerham C. C. Weir B. W. (1984). Covariances of relatives stemming from a population undergoing mixed self and random mating. Biometrics 40, 157–164. 10.2307/2530754 6733226

[B44] Cubero J. (1973). Evolutionary trends in *Vicia faba* L. Theor. Appl. Genet. 43, 59–65. 10.1007/BF00274958 24424894

[B45] Cubero J. Suso M. J. (1981). Primitive and modern forms of *Vicia faba*. Kulturpflanze 29:137. 10.1007/BF02014744

[B46] Cubero J. I. (1974). On the evolution of *Vicia faba* L. Theor. Appl. Genet. 45, 47–51. 10.1007/BF00283475 24419274

[B47] Cubero J. I. (1994). Breeding work in Spain for Orobanche resistance in faba bean and sunflower, in Biology and Management of Orobanche, Proceedings of the Third International Workshop on Orobanche and Related Striga Research, eds Pieterse A. H. Verkleij J. A. C. ter Borg S. J. (Amsterdam: Royal Tropical Institute), 465–473.

[B48] Del Papa M. F. Balagué L. J. Sowinski S. C. Wegener C. Segundo E. Abarca F. M. . (1999). Isolation and characterization of alfalfa-nodulating rhizobia present in acidic soils of central Argentina and Uruguay. Appl. Environ. Microbiol. 65:1420. 10.1128/AEM.65.4.1420-1427.1999 10103231 PMC91201

[B49] Díaz-Ruiz R. Satovic Z. Ávila C. M. Alfaro C. M. Gutierrez M. V. Torres A. M. . (2009). Confirmation of QTLs controlling *Ascochyta fabae* resistance in different generations of faba bean (*Vicia faba* L.). Crop Pasture Sci. 60, 353–361. 10.1071/CP08190

[B50] Dieckmann S. Link W. (2010). Quantitative genetic analysis of embryo heterosis in faba bean (*Vicia faba* L.). Theor. Appl. Genet. 120, 261–270. 10.1007/s00122-009-1057-7 19449175 PMC2793387

[B51] Drayner J. M. (1956). Regulation of outbreeding in field beans (*Vicia faba*). Nature 177, 489–490. 10.1038/177489b0

[B52] Duc G. (1995). Mutagenesis of faba bean (*Vicia faba* L.) and the identification of five different genes controlling no nodulation, ineffective nodulation or supernodulation. Euphytica 83, 147–152. 10.1007/BF01678042

[B53] Duc G. (1997). Faba bean (*Vicia faba* L.). Field Crops Res. 53, 99–109.

[B54] Duc G. Bao S. Baum M. Redden B. Sadiki M. Suso M. J. . (2010). Diversity maintenance and use of *Vicia faba* L. genetic resources. Field Crops Res. 115, 270–278. 10.1016/j.fcr.2008.10.003

[B55] Duc G. Le Guen J. Picard J. Berthelem P. (1985). Proposed use for a newly Y-discovered dominant male sterile allele for breeding purposes in *Vicia faba*. FABIS Newsl. 12, 8–10.

[B56] Duc G. Marget P. Esnault R. Le Guen J. Bastianelli D. (1999). Genetic variability for feeding value of faba bean seeds (*Vicia faba*): comparative chemical composition of isogenics involving zero-tannin and zero-vicine genes. J. Agric. Sci. 133, 185–196. 10.1017/S0021859699006905

[B57] Duc G. Sixdenier G. Lila M. Furstoss V. (1989). Search of genetic variability for vicine and convicine content in *Vicia faba* L., in Proceedings of the Conference of a First Report of a Gene which Codes for Nearly Zero-Vicine and Zero-Convicine Contents (Wageningen), 305–313.

[B58] Duc G. Stoddard F. (2018). David Bond and Jean Picard: two pivotal breeders of faba bean in the 20th century. Plant Genet. Resour. 16, 483–487. 10.1017/S147926211800031X

[B59] Ederer W. Link W. (1992b). The Polycross-test as step in breeding partially allogamous crops. Theoretical considerations, in XIIIth EUCARPIA Congress, Book of Abstracts, France (Angers: EUCARPIA), 543–544.

[B60] Ederer W. Link W. (1992a). How shall we select the components for open pollinated faba bean (*Vicia faba* L.) varieties?, in Proc. 1st European Conference on Grain Legumes, Angers, France (Paris: Association Européenne des Protéagineux), 71–72.

[B61] Ederer W. Link W. (1993). The concept of varietal ability for partially allogamous crops. Plant Breed. 110, 1–8. 10.1111/j.1439-0523.1993.tb00561.x

[B62] Falconer D. S. Mackay T. F. C. (1996). Introduction to Quantitative Genetics, 4th Edn, Harlow: Addison Wesley Longman.

[B63] FAOSTAT (2021). Food and Agriculture Organization of the United Nations. Available online at: http://faostat.fao.org (accessed on June 7, 2021).

[B64] Faridi R. Koopman B. Schierholt A. Ali M. B. Apel S. Link W. (2021). Genetic study of the resistance of faba bean (*Vicia faba*) against the fungus *Ascochyta fabae* through a genome-wide association analysis. Plant Breed. 140, 442–452. 10.1111/pbr.12918

[B65] Farooq M. Gogoi N. Hussain M. Barthakur S. Paul S. Bharadwaj N. . (2017). Effects, tolerance mechanisms and management of salt stress in grain legumes. Plant Physiol. Biochem. 118, 199–217. 10.1016/j.plaphy.2017.06.020 28648997

[B66] Fasoula V. Tokatlidis I. (2012). Development of crop cultivars by honeycomb breeding. Agron. Sustain. Dev. 32, 161–180. 10.1007/s13593-011-0034-0

[B67] Fernández-Aparicio M. Flores F. Rubiales D. (2016). The Effect of Orobanche crenata Infection Severity in Faba Bean, Field Pea, and Grass Pea Productivity. Front Plant Sci. 7:1409. 10.3389/fpls.2016.01409 27708660 PMC5030276

[B68] Fleck A. Ruckenbauer P. (1989). Der Polycrosstest als methodischer Schritt in der Fababohnenzüchtung (experimentelle Ergebnisse). Bodenkultur 40, 61–72.

[B69] Gasim S. Abel S. Link W. (2004). Extent, variation and breeding impact of natural cross-fertilization in German winter faba beans using hilum colour as marker. Euphytica 136, 193–200. 10.1023/B:EUPH.0000030669.75809.dc

[B70] Gaynor R. C. Gorjanc G. Bentley A. R. Ober E. S. Howell P. Jackson R. . (2017). A two-part strategy for using genomic selection to develop Inbred lines. Crop Sci. 57, 2372–2386. 10.2135/cropsci2016.09.0742

[B71] Ghaouti L. Link W. (2009). Local vs. formal breeding and inbred line vs. synthetic cultivar for organic farming: case of Vicia faba L. Field Crops Res. 110, 167–172. 10.1016/j.fcr.2008.07.013

[B72] Ghaouti L. Vogt-Kaute W. Link W. (2008). Development of locally-adapted faba bean cultivars for organic conditions in Germany through a participatory breeding approach. Euphytica 162, 257–268. 10.1007/s10681-007-9603-3

[B73] Gharzeddin K. Maalouf F. Khoury B. Khater L. A. Christmann S. El Dine N. A. J. (2019). Efficiency of different breeding strategies in improving the faba bean productivity for sustainable agriculture. Euphytica 215:203. 10.1007/s10681-019-2521-3

[B74] Gorfu D. (1996). Morphological, cultural and pathogenic variability among nine isolates of *Botrytis fabae* from Ethiopia. FABIS Newsl. 38-39, 37–41.

[B75] Gorfu D. Yaynu H. (2001). Yield loss of crops due to plant diseases in Ethiopia. Pest Manag. J. Ethiopia 5, 55–67.

[B76] Graham P. H. (1992). Stress tolerance in *Rhizobium* and *Bradyrhizobium*, and nodulation under adverse soil conditions. Can. J. Microbiol. 38, 475–484. 10.1139/m92-079

[B77] Gressel J. Hanafi A. Head G. Marasas W. Obilana B. Ochanda J. . (2004). Major heretofore intractable biotic constraints to African food security that may be amenable to novel biotechnological solutions. Crop Prot. 23, 661–689. 10.1016/j.cropro.2003.11.014

[B78] Gutierrez N. Avila C. M. Torres A. M. (2020). The bHLH transcription factor *VfTT8* underlies *zt2*, the locus determining zero tannin content in faba bean (*Vicia faba* L.). Sci. Rep. 10:14299. 10.1038/s41598-020-71070-2 32868815 PMC7459296

[B79] Gutierrez N. Torres A. M. (2019). Characterization and diagnostic marker for *TTG1* regulating tannin and anthocyanin biosynthesis in faba bean. Sci. Rep. 9:16174. 10.1038/s41598-019-52575-x 31700069 PMC6838129

[B80] Hampton G. (1980). The significance of Ascochyta fabae in broad beans in the Manawatu, and methods for its control. N. Z. J. Crop Hortic. Sci. 8, 305–308. 10.1080/03015521.1980.10426279

[B81] Hanounik S. B. Maliha N. (1986). Horizontal and vertical resistance in *Vicia faba* to chocolate spot caused by *Botrytis fabae*. Plant Dis. 70, 770–773. 10.1094/PD-70-770

[B82] Hanounik S. B. Robertson L. D. (1988). New sources of resistance in *Vicia faba* to chocolate spot caused by *Botrytis fabae*. Plant Dis. 72, 696–698. 10.1094/PD-72-0696

[B83] Hanounik S. B. Robertson L. D. (1989). Resistance in *Vicia faba* germplasm to blight caused by *Ascochyta fabae*. Plant Dis. 73, 202–205. 10.1094/PD-73-0202

[B84] Hauggaard-Nielsen H. Mundus S. Jensen E. S. (2009). Nitrogen dynamics following grain legumes and subsequent catch crops and the effects on succeeding cereal crops. Nutr. Cycling Agroecosyst. 84, 281–291. 10.1007/s10705-008-9242-7

[B85] Herath I. H. M. H. B. (1997). Genetic studies on faba bean rust and rust resistance on faba bean (MSc thesis). The University of Sydney, Sydney, Australia.

[B86] Herridge D. F. Peoples M. B. Boddey R. M. (2008). Global inputs of biological nitrogen fixation in agricultural systems. Plant Soil 311, 1–18. 10.1007/s11104-008-9668-3

[B87] Horn L. N. Ghebrehiwot H. M. Shimelis H. A. (2016). Selection of novel cowpea genotypes derived through gamma irradiation. Front. Plant Sci. 7:262. 10.3389/fpls.2016.00262 27148275 PMC4834446

[B88] Howieson J. G. Ewing M. A. D'antuono M. F. (1988). Selection for acid tolerance in Rhizobium meliloti. Plant Soil 105, 179–188.

[B89] Huyghe C. (1998). Genetics and genetic modifications of plant architecture in grain legumes: a review. Agronomie 18, 383–411. 10.1051/agro:19980505

[B90] Ijaz U. Adhikari K. Kimber R. Trethowan R. Bariana H. Bansal U. (2021a). Pathogenic specialization in *Uromyces viciae-fabae* in Australia and rust resistance in faba bean. Plant Dis. 105, 636–642. 10.1094/PDIS-06-20-1325-RE 32852254

[B91] Ijaz U. Sudheesh S. Kaur S. Sadeque A. Bariana H. Bansal U. . (2021b). Mapping of two new rust resistance genes *Uvf-2* and *Uvf-3* in faba bean. Agronomy 11:1370. 10.3390/agronomy11071370

[B92] Inci N. E. Toker C. (2011). Screening and selection of faba beans (*Vicia faba* L.) for cold tolerance and comparison to wild relatives. Genet. Resour. Crop Evol. 58, 1169–1175. 10.1007/s10722-010-9649-2

[B93] International Atomic Energy Agency (2021). Available online at: https://bit.ly/3yVEReM (accessed on September 9, 2021).

[B94] Jacquier N. M. A. Gilles L. M. Pyott D. E. Martinant J.-P. Rogowsky P. M. Widiez T. (2020). Puzzling out plant reproduction by haploid induction for innovations in plant breeding. Nature Plants 6, 610–619. 10.1038/s41477-020-0664-9 32514145

[B95] Jida M. Assefa F. (2014). Effects of acidity on growth and symbiotic performance of rhizobium leguminosarum bv. viciae strains isolated from faba bean producing areas of Ethiopia. Sci. Technol. Arts Res. J. 3, 26–33. 10.4314/star.v3i2.4

[B96] Karppinen E. M. Payment J. Chatterton S. Bainard J. D. Hubbard M. Gan Y. . (2020). Distribution and abundance of Aphanomyces euteiches in agricultural soils: effect of land use type, soil properties, and crop management practices. Appl. Soil Ecol. 150:103470. 10.1016/j.apsoil.2019.103470

[B97] Kassa Y. Ayele T. Worku Y. Teferra B. (2020). Participatory evaluation of faba bean gall disease (*Olpidium viciae*) management options in the highland disease hotspot areas of South-Eastern Amhara Region, Ethiopia: an integrated approach. Cogent Food Agri. 6:1801216. 10.1080/23311932.2020.1801216

[B98] Kaur S. Kimber R. B. E. Cogan N. O. I. Materne M. Forster J. W. Paull J. G. (2014). SNP discovery and high-density genetic mapping in faba bean (*Vicia faba* L.) permits identification of QTLs for ascochyta blight resistance. Plant Sci. 217-218, 47–55. 10.1016/j.plantsci.2013.11.014 24467895

[B99] Keneni A. Prabu P. C. Assefa F. (2010). Characterization of acid and salt tolerant rhizobial strains isolated from faba bean fields of Wollo, Northern Ethiopia. J. Agric. Sci. Technol. 12, 365–376.

[B100] Khalil S. Kharrat M. Malhotra R. Saxena M. Erskine W. (2004). Breeding faba bean for Orobanche resistance, in Proceedings of the Expert Consultation on IPM for Orobanche in Food Legume Systems in the Near East and North Africa, Integrated Management of Orobanche in Food Legumes in the Near East and North Africa, eds Dahan R. El-Mourid M. (Rabat), 1–18.

[B101] Khan H. R. Link W. Hocking T. J. Stoddard F. L. (2007). Evaluation of physiological traits for improving drought tolerance in faba bean (*Vicia faba* L.). Plant Soil 292, 205–217. 10.1007/s11104-007-9217-5

[B102] Khazaei H. Link W. Street K. Stoddard F. L. (2018a). ILB 938, a valuable faba bean (*Vicia faba* L.) accession. Plant Genet. Res. 16, 478–482. 10.1017/S1479262118000205

[B103] Khazaei H. Mäkelä P. S. Stoddard F. L. (2018b). Ion beam irradiation mutagenesis in rye (*Secale cereale* L.), linseed (*Linum usitatissimum* L.) and faba bean (*Vicia faba* L.). Agric. Food Sci. 27, 146–151. 10.23986/afsci.70780

[B104] Khazaei H. O'Sullivan D. M. Jones H. Pitts N. Sillanpää M. J. Pärssinen P. . (2015). Flanking SNP markers for vicine-convicine concentration in faba bean (*Vicia faba* L.). Mol. Breed. 35:38. 10.1007/s11032-015-0214-8

[B105] Khazaei H. O'Sullivan D. M. Stoddard F. L. Adhikari K. N. Paull J. G. Schulman A. H. . (2021). Recent advances in faba bean genetic and genomic tools for crop improvement. Legum. Sci. (in press). 10.1002/leg3.75 PMC8700193 34977588

[B106] Khazaei H. Purves R. W. Hughes J. Link W. O'Sullivan D. M. Schulman A. H. . (2019a). Eliminating vicine and convicine, the main anti-nutritional factors restricting faba bean usage. Trends Food Sci. Technol. 91, 549–556. 10.1016/j.tifs.2019.07.051

[B107] Khazaei H. Purves R. W. Song M. Stonehouse R. Bett K. E. Stoddard F. L. . (2017). Development and validation of a robust, breeder-friendly molecular marker for the *vc*^−^ locus in faba bean. Mol. Breed. 37:140. 10.1007/s11032-017-0742-5

[B108] Khazaei H. Street K. Santanen A. Bari A. Stoddard F. L. (2013). Do faba bean (*Vicia faba* L.) accessions from environments with contrasting seasonal moisture availabilities differ in stomatal characteristics and related traits? Genet. Res. Crop Evol. 60, 2343–2357. 10.1007/s10722-013-0002-4

[B109] Khazaei H. Subedi M. Nickerson M. Martínez-Villaluenga C. Frias J. Vandenberg A. (2019b). Seed protein of lentils: current status, progress, and food applications. Foods 8:391. 10.3390/foods8090391 31487958 PMC6769807

[B110] Khursheed S. Raina A. Laskar R. A. Khan S. (2018). Effect of gamma radiation and EMS on mutation rate: their effectiveness and efficiency in faba bean (*Vicia faba* L.). Caryologia 71, 397–404. 10.1080/00087114.2018.1485430

[B111] Khursheed S. Raina A. Parveen K. Khan S. (2019). Induced phenotypic diversity in the mutagenized populations of faba bean using physical and chemical mutagenesis. J. Saudi Soc. Agric. Sci. 18, 113–119. 10.1016/j.jssas.2017.03.001

[B112] Kohpina S. Knight R. Stoddard F. L. (2000). Genetics of resistance to a*scochyta blight* in two populations of faba bean. Euphytica 112, 101–107. 10.1023/A:1003853126862

[B113] Kumari S. G. Makkouk K. M. (2007). Virus diseases of faba bean (*Vicia faba* L.) in Asia and Africa. Plant Viruses 1, 93–105.

[B114] Ladizinsky G. (1975). Seed protein electrophoresis of the wild and cultivated species of selection *faba* of *Vicia*. Euphytica 24, 785–788. 10.1007/BF00132919

[B115] Landry E. J. Coyne C. J. McGee R. J. Hu J. (2017). A modified mass selection scheme for creating winter-hardy faba bean (*Vicia faba* L.) lines with a broad genetic base. Euphytica 213:72. 10.1007/s10681-017-1843-2

[B116] Link W. (1990). Autofertility and rate of cross-fertilization: crucial characters for breeding synthetic varieties in faba beans (*Vicia faba* L.). Theor. Appl. Genet. 79, 713–717. 10.1007/BF00226888 24226589

[B117] Link W. (1995). Exploitation of hybrid vigour in faba bean (*Vicia faba* L.), in Proceedings of the 2nd European Conference on Grain Legumes (Copenhagen), 218–219.

[B118] Link W. (1998). Neues über CMS bei Ackerbohnen, in Arbeitstagung der Arbeitsgemeinschaft der Saatzuchtleiter (Gumpenstein), 25. bis 27, 95–101.

[B119] Link W. (2009). Züchtungsforschung bei der Ackerbohne: Fakten und Potentiale. J. Kulturpflanzen 61, 341–347. 10.5073/JfK.2009.09.08

[B120] Link W. Abdelmula A. A. Kittlitz E. V. Bruns S. Riemer H. Stelling D. (1999). Genotypic variation for drought tolerance in *Vicia faba*. Plant Breed. 118, 477–484. 10.1046/j.1439-0523.1999.00412.x

[B121] Link W. Balko C. Stoddard F. L. (2010). Winter hardiness in faba bean: physiology and breeding. Field Crops Res. 115, 287–296. 10.1016/j.fcr.2008.08.004

[B122] Link W. Ederer W. Gumber R. K. Melchinger A. E. (1997). Detection and characterization of two new CMS systems in faba bean (*Vicia faba*). Plant Breed. 116, 158–162. 10.1111/j.1439-0523.1997.tb02171.x

[B123] Link W. Ederer W. Metz P. Buiel H. Melchinger A. E. (1994). Genotypic and environmental variation of degree of cross-fertilization in faba bean. Crop Sci. 34, 960–964. 10.2135/cropsci1994.0011183X003400040024x

[B124] Link W. Ederer W. Ruckenbauer P. (1991). Züchtung von synthetischen Sorten bei der Fababohne: detaillierte Untersuchungen über die Zuchtmethode auf experimentellem und theoretischem Gebiet. Bundesministerium für Ernährung, Landwirtschaft und Forsten, Forschungsdokumentation Nachwachsende Rohstoffe, 253–262.

[B125] Link W. Stützel H. (1995). Faba bean. Genetics, in Advances in Plant Breeding. Physiological Potentials for Yield Improvement of Annual Oil and Protein Crops, Vol. 17, eds Diepenbrock W. Becker H. C. (Berlin: Blackwell), 239–278.

[B126] Liu H. Tessema B. B. Jensen J. Cericola F. Andersen J. R. Sørensen A. C. (2019). ADAM-Plant: a software for stochastic simulations of plant breeding from molecular to phenotypic level and from simple selection to complex speed breeding programs. Front. Plant Sci. 9:1926. 10.3389/fpls.2018.01926 30687343 PMC6333911

[B127] Maalouf F. Ahmed S. Shaaban K. Bassam B. Nawar F. Singh M. . (2016). New faba bean germplasm with multiple resistances to Ascochyta blight, chocolate spot and rust diseases. Euphytica 211, 157–167. 10.1007/s10681-016-1726-y

[B128] Maalouf F. Hu J. O'Sullivan D. M. Zong Z. Hamwieh A. Kumar S. . (2019). Breeding and genomics status in faba bean (*Vicia faba*). Plant Breed. 138, 465–473. 10.1111/pbr.12644

[B129] Maalouf F. Khalil S. Ahmed S. Kharrat M. Hajjar S. El Shama'a K. (2011). Yield stability of faba bean lines under diverse broomrape prone production environments. Field Crops Res. 124, 288–294. 10.1016/j.fcr.2011.06.005

[B130] Maalouf F. Patil S. Kemal S. A. (2018). Developing improved varieties of faba bean, in Achieving Sustainable Cultivation of Grain Legumes Volume 2: Improving Cultivation of Particular Grain Legumes. Burleigh Dodds Science. Available online at: https://repo.mel.cgiar.org/handle/20.500.11766/6402 (accessed August 12, 2021).

[B131] Maalouf F. S. Suso M. J. Moreno M. T. (1999). Choice of methods and indices for identifying the best parentals for synthetic varieties in faba bean. Agronomie 19, 705–712. 10.1051/agro:19990805

[B132] Mao D. Michelmore S. Paull J. Preston C. Sutton T. Oldach K. . (2019). Phenotypic and molecular characterisation of novel *Vicia faba* germplasm with tolerance to acetohydroxyacid synthase-inhibiting herbicides (AHAS) developed through mutagenesis techniques. Pest Manag. Sci. 75, 2698–2705. 10.1002/ps.5378 30779284

[B133] Maqbool A. Shafiq S. Lake L. (2010). Radiant frost tolerance in pulse crops—a review. Euphytica 172, 1–12. 10.1007/s10681-009-0031-4

[B134] Marcellos H. Moore K. J. Nikandrow A. (1995). Influence of foliar-applied fungicides on seed yield of faba bean (*Vicia faba* L.) in northern New South Wales. Aust. J. Exp. Agric. 35, 97–102. 10.1071/EA9950097

[B135] Marzinzig B. Brünjes L. Biagioni S. Behling H. Link W. Westphal C. (2018). Bee pollinators of faba bean (*Vicia faba* L.) differ in their foraging behaviour and pollination efficiency. Agric. Ecosyst. Environ. 264, 24–33. 10.1016/j.agee.2018.05.003

[B136] Maxted N. Callimassia M. A. Bennett M. D. (1991). Cytotaxonomic studies of Eastern Mediterranean *Vicia* species (Leguminosae). Plant Syst. Evol. 177, 221–234. 10.1007/BF00937959

[B137] Mejri S. Mabrouk Y. Belhadj O. Saidi M. (2018). Orobanche foetida resistance in two new faba bean genotypes produced by radiation mutagenesis. Int. J. Radiat. Biol. 94, 671–677. 10.1080/09553002.2018.1486517 29893613

[B138] Micke A. (1984). Mutation breeding of grain legumes. Plant Soil. 152, 81–85.

[B139] Mills A. M. Allsman L. A. Leon S. Rasmussen C. G. (2020). Using seed chipping to genotype maize kernels. Bio 101:e3553. 10.21769/BioProtoc.3553

[B140] Mobini S. Khazaei H. Warkentin T. D. Vandenberg A. (2020). Shortening the generation cycle in faba bean (*Vicia faba*) by application of cytokinin and cold stress to assist speed breeding. Plant Breed. 139, 1181–1189. 10.1111/pbr.12868

[B141] Moehring J. Williams E. R. Piepho H.-P. (2013). Efficiency of augmented p-rep designs in multi-environmental trials. Theor. Appl. Genet. 127, 1049–1060. 10.1007/s00122-014-2278-y 24553963

[B142] Muktadir M. A. (2009). Understanding morphological, physiological and biochemical characteristics of faba bean (Vicia faba L.) under drought condition (Ph.D. thesis). University of Sydney, Sydney, Australia. Available online at: http://hdl.handle.net/2123/20656

[B143] Muktadir M. A. Adhikari K. N. Ahmad N. Merchant A. (2021). Chemical composition and reproductive functionality of contrasting faba bean genotypes in response to water deficit. Physiol. Plant. 172, 540–551. 10.1111/ppl.13309 33305355

[B144] Müller D. (2017). Genomic selection in synthetic populations (Ph.D. thesis). University of Hohenheim, Stuttgart, Germany.

[B145] Mulugeta W. Tesfaye K. Getnet M. Ahmed S. Nebiyu A. Mekuanint F. (2019). Quantifying yield potential and yield gaps of faba bean in Ethiopia. Ethiop. J. Agric. Sci. 29, 105–120.

[B146] Nurmansyah Alghamdi S. S. Migdadi H. H. (2020). Morphological diversity of faba bean (*Vicia faba* L.) M2 mutant populations induced by gamma radiation and diethyl sulfate. J. King Saud Univ. Sci. 32, 1647–1658. 10.1016/j.jksus.2019.12.024

[B147] Nurmansyah Alghamdi S. S. Migdadi H. M. Farooq M. (2019). Novel inflorescence architecture in gamma radiation-induced faba bean mutant populations. Int. J. Radiat. Biol. 95, 1744–1751. 10.1080/09553002.2019.1665205 31486707

[B148] Olszewski A. (1996). Optimierung der Frostschadensbestimmung mit Hilfe der Chlorophyll-Fuoreszenz-Methode und deren Einsatz für ein Frostresistenzscreening bei *Vicia faba* L. (Optimization of frost-injury assessment via chlorophyll-fluoresence and employment to screen for frost resistance in *Vicia faba* L.), 127.

[B149] Pérez-de-Luque A. Eizenberg H. Grenz J. H. Sillero J. C. Ávila C. M. Sauerborn J. . (2010). Broomrape management in faba bean. Field Crops Res. 115, 319–328. 10.1016/j.fcr.2009.02.013

[B150] Pfeiffer P. Jung J. L. Heitzler J. Keith G. (1993). Unusual structure of the double-stranded RNA associated with the “447” cytoplasmic male sterility in *Vicia faba*. J. Gen. Virol. 74, 1167–1173. 10.1099/0022-1317-74-6-1167 7685375

[B151] Pulse Australia (2021). Available online at: https://www.pulseaus.com.au/growing-pulses/bmp/faba-and-broad-bean (accessed on August 27, 2021).

[B152] Raina A. Khan S. (2020). Mutagenic effectiveness and efficiency of gamma rays and sodium azide in M_2_ generation of Cowpea [*Vigna unguiculata* (L.) Walp.]. bioRxiv 2020:983486. 10.1101/2020.03.09.983486

[B153] Raina S. Rees H. (1983). DNA variation between and within chromosome complements of *Vicia* species. Heredity 51, 335–346. 10.1038/hdy.1983.38

[B154] Ramsay G. Griffiths D. W. Dow N. D. (1991). Spontaneous and induced variation in levels of vicine and convicine in faba beans. Asp. Appl. Biol. 27, 43–47.

[B155] Ramsay G. Pickersgill B. (1986). Interspecific hybridization between *Vicia faba* and other species of *Vicia*: approaches to delaying embryo abortion. Biol. Zentralbl. 105, 171–189.

[B156] Rashid K. Y. Bernier C. C. (1986). Selection for slow rusting in faba bean (*Vicia faba* L.) to *Uromyces viciae-fabae*. Crop Prot. 5, 218–224. 10.1016/0261-2194(86)90106-7

[B157] Rashid K. Y. Bernier C. C. (1991). The effect of rust on yield of faba bean cultivars and slow-rusting populations. Can. J. Plant Sci. 71, 967–972. 10.4141/cjps91-139

[B158] Román B. Satovic Z. Avila C. M. Rubiales D. Moreno M. T. Torres A. M. (2003). Locating genes associated with *Ascochyta fabae* resistance in *Vicia faba*. Aust. J. Agric. Res. 54, 85–90. 10.1071/AR02034

[B159] Rubiales D. Fernández-Aparicio M. (2012). Innovations in parasitic weeds management in legume crops. A review. Agron. Sustain. Dev. 32, 433–449. 10.1007/s13593-011-0045-x

[B160] Rubiales D. Flores F. Emeran A. S. Kharrat M. Amri M. Rojas-Molina M. M. . (2014). Identification and multi-environment validation of resistance against broomrapes (*Orobanche crenata* and *O. foetida) in faba bean (Vicia faba)*. Field Crops Res. 166, 58–65. 10.1016/j.fcr.2014.06.010

[B161] Rubiales D. Rojas-Molina M. M. Sillero J. C. (2016). Characterization of Resistance Mechanisms in Faba Bean (*Vicia faba*) against Broomrape Species (*Orobanche* and *Phelipanche* spp.). Front. Plant Sci. 7:1747. 10.3389/fpls.2016.01747 27920790 PMC5118618

[B162] Sallam A. Martsch R. Moursi Y. S. (2015). Genetic variation in morpho-physiological traits associated with frost tolerance in faba bean (*Vicia faba* L.). Euphytica 205, 395–408. 10.1007/s10681-015-1395-2

[B163] Samineni S. Sen M. Sajja S. B. Gaur P. M. (2020). Rapid generation advance (RGA) in chickpea to produce up to seven generations per year and enable speed breeding. Crop J. 8, 164–169. 10.1016/j.cj.2019.08.003

[B164] Saxena M. C. (1991). Status and scope for production of faba bean in the Mediterra-nean countries. *Options Mediterr*. Serie A 10, 15–20.

[B165] Schill B. Bund A. Glass C. Link W. (1992). Factors determining the performance of synthetics in faba beans, in Experimentals Results. XIIIrd EUCARPIA Congress, Book of Poster Abstracts (Angers: EUCARPIA), 585–586.

[B166] Siddique K. H. M. Regan K. L. Tennant D. Tomson B. D. (2001). Water use and water use efficiency of cool season grain legumes in low rainfall Mediterranean-type environments. Eur. J. Agron. 15, 267–280. 10.1016/S1161-0301(01)00106-X

[B167] Sillero J. C. Villegas-Fernandez A. M. Thomas J. Rojas-Molina M. M. Emeran A. A. Fernandez-Aparicio M. . (2010). Faba bean breeding for disease resistance. Field Crops Res. 115, 297–307. 10.1016/j.fcr.2009.09.012

[B168] Sjödin J. (1971). Induced morphological variation in *Vicia faba* L. Hereditas 67, 155–180. 10.1111/j.1601-5223.1971.tb02371.x

[B169] Stoddard F. L. Balko C. Erskine W. Khan H. R. Link W. Sarker A. (2006). Screening techniques and sources of resistance to abiotic stresses in cool-season food legumes. Euphytica 147, 167–186. 10.1007/s10681-006-4723-8

[B170] Stoddard F. L. Kohpina S. Knight R. (1999). Variability of *Ascochyta fabae* in South Australia. Aust. J. Agric. Res. 50, 1475–1481. 10.1071/AR98204

[B171] Sudheesh S. Kimber R. B. E. Braich S. Forster J. W. Paull J. G. Kaur S. (2019). Construction of an integrated genetic linkage map and detection of quantitative trait loci for ascochyta blight resistance in faba bean (*Vicia faba* L.). Euphytica 215:42. 10.1007/s10681-019-2365-x

[B172] Suso M. J. Bebeli P. J. Christmann S. Mateus C. Negri V. Pinheiro de Carvalho M. A. A. . (2016). Enhancing legume ecosystem services through an understanding of plant–pollinator interplay. Front. Plant Sci. 7:333. 10.3389/fpls.2016.00333 27047514 PMC4796003

[B173] Suso M. J. Moreno M. T. Melchinger A. E. (1999). Variation in outcrossing rate and genetic structure on six cultivars of *Vicia faba* L. as affected by geographic location and year. Plant Breed. 118, 347–350. 10.1046/j.1439-0523.1999.00389.x

[B174] Suso M. J. Pierre J. Moreno M. T. Esnault R. Le Guen J. (2001). Variation in outcrossing levels in faba bean cultivars: role of ecological factors. J. Agri. Sci. 136, 399–405. 10.1017/S0021859601008851

[B175] Tacke R. Ecke W. Höfer M. Sass O. Link W. (2021). Zooming into the genomic vicinity of the major locus for vicine and convicine in faba bean (*Vicia fab*a L.). bioRxiv. 10.1101/2021.02.19.431996

[B176] Tadege M. Wang T. L. Wen J. Ratet P. Mysore K. S. (2009). Mutagenesis and beyond! Tools for understanding legume biology. Plant Physiol. 151, 978–984. 10.1104/pp.109.144097 19741047 PMC2773078

[B177] Tagkouli V. (2020). Evaluation of recurrent selection as a method to achieve rapid re-adaptation of faba bean to a niche agro-climate (Ph.D. thesis). University of Reading, Reading, UK.

[B178] Tavakkoli E. Paull J. Rengasamy P. McDonald G. K. (2012). Comparing genotypic variation in faba bean (*Vicia faba* L.) in response to salinity in hydroponic and field experiments. Field Crops Res. 127, 99–108. 10.1016/j.fcr.2011.10.016

[B179] Temesgen T. Keneni G. Sefera T. Jarso M. (2015). Yield stability and relationships among stability parameters in faba bean (*Vicia faba* L.) genotypes. Crop J. 3, 258–269. 10.1016/j.cj.2015.03.004

[B180] Toker C. Mutlu N. (2011). Breeding for abiotic stresses, in Biology and Breeding of Food Legumes, eds Pratap A. Kumar J. (Wallingford: CABI Publications), 241-261. 10.1079/9781845937669.0241

[B181] Tolessa T. T. Keneni G. Mohammad H. (2015). Genetic progresses from over three decades of faba bean (*Vicia faba* L.) breeding in Ethiopia. Aust. J. Crop Sci. 9, 41–48. Available online at: http://www.cropj.com/tolessa_9_1_2015_41_48.pdf

[B182] Torres A. M. Moreno M. T. Cubero J. I. (1993). Genetics of six components of autofertility in *Vicia faba*. Plant Breed. 110, 220–228. 10.1111/j.1439-0523.1993.tb00581.x

[B183] van Leur J. A. G. Kumari S. G. Makkouk K. M. Rose I. A. (2006). Viruses on fababean in North-East Australia and strategies for virus control, in Proceeings of the International Workshop on Faba Bean Breeding and Agronomy, eds Avila C. M. Cubero J. I. Moreno M. T. Suso M. J. Torres A. M. 129–131.

[B184] van Norel A. Hoogendoorn J. (1989). Faba bean dwarf selections outyield Dutch top varieties, FABIS Newsl. 25, 10–13.

[B185] Wang H.-F. Zong X.-X. Guan J.-P. Yang T. Sun X.-L. Ma Y. Redden R. (2012). Genetic diversity and relationship of global faba bean (*Vicia faba* L.) germplasm revealed by ISSR markers. Theor. Appl. Genet. 124, 789–797. 10.1007/s00122-011-1750-1 22204023

[B186] Warsame A. O. Michael N. O'Sullivan D. M. Tosi P. (2020). Identification and quantification of major faba bean seed proteins. J. Agri Food Chem. 68, 8535–8544. 10.1021/acs.jafc.0c02927 32678595 PMC7458416

[B187] Webb A. Cottage A. Wood T. Khamassi K. Hobbs D. Gostkiewicz K. . (2016). A SNP-based consensus genetic map for synteny-based trait targeting in faba bean (*Vicia faba* L.). Plant Biotechnol. J. 14, 177–185. 10.1111/pbi.12371 25865502 PMC4973813

[B188] Wijaya A. (2003). Towards Interspecific Hybridization in Vicia faba L. Göttingen: Cuvillier Verlag.

[B189] Wondwosen W. Dejene M. Tadesse N. Ahmed S. (2019). Evaluation of faba bean (*Vicia faba* L.) varieties against faba bean gall disease in north Shewa zone, Ethiopia. Rev. Plant Stud. 6, 11–20. 10.18488/journal.69.2019.61.11.20

[B190] World Bank Group (2019). Sustainable Land Management And Restoration in the Middle East and North Africa Region: Issues, Challenges, and Recommendations. Washington, DC: World Bank Group. Available online at: http://hdl.handle.net/10986/33037

[B191] Yitayih G. Azmeraw Y. (2017). Adaptation of faba bean varieties for yield, for yield components and against faba bean gall (*Olpidium viciae Kusano*) disease in South Gondar, Ethiopia. Crop J. 5, 560–566, 10.1016/j.cj.2017.05.007

[B192] You M. P. Eshete B. B. Kemal S. A. van Leur J. Barbetti M. J. (2021). *Physoderma*, not *Olpidium*, is the true cause of faba bean gall disease of *Vicia faba* in Ethiopia. Plant Pathol. 70, 1180–1194. 10.1111/ppa.13359

[B193] Zahran H. H. (1999). Rhizobium-legume symbiosis and nitrogen fixation under severe conditions and in an arid climate. Microbiol. Mol. Biol. Rev. 63:968. 10.1128/MMBR.63.4.968-989.1999 10585971 PMC98982

[B194] Zahran M. K. El Nasr Ibrahim T. S. Farag F. H. Korollos M. A. (1980). Chemical control of Orobanche crenata in *Vicia faba*. FABIS Newsl. 2, 47–49.

[B195] Zanotto S. Vandenberg A. Khazaei H. (2020). Development and validation of a robust KASP marker for *zt2* locus in faba bean (*Vicia faba*). Plant Breed. 139, 375–380. 10.1111/pbr.12772

[B196] Zeid M. Schön C. C. Link W. (2004). Hybrid performance and AFLP-based genetic similarity in faba bean. Euphytica 139, 207–216. 10.1007/s10681-004-3156-5

[B197] Zenkteler M. Tegeder M. Schieder O. (1998). Embryological studies on reciprocal crosses between *Vicia faba* and *Vicia narbonensis*. Acta Soc. Bot. Pol. 67, 37–43. 10.5586/asbp.1998.004

[B198] Zhou R. Hyldgaard B. Yu X. Rosenqvist E. Ugarte R. M. Yu S. . (2018). Phenotyping of faba beans (*Vicia faba* L.) under cold and heat stresses using chlorophyll fluorescence. Euphytica 214, 1–13. 10.1007/s10681-018-2154-y

[B199] Zong X. Liu X. Guan J. Wang S. Liu Q. Paull J. G. . (2009). Molecular variation among Chinese and global winter faba bean germplasm. Theor. Appl. Genet. 118, 971–978. 10.1007/s00122-008-0954-5 19169661

[B200] Zong X. Ren J. Guan J. Wang S. Liu Q. Paull J. G. . (2010). Molecular variation among Chinese and global germplasm in spring faba bean areas. Plant Breed. 129, 508–513. 10.1111/j.1439-0523.2009.01718.x

