# Supplementary material for: Conventional and Molecular Breeding Tools for Accelerating Genetic Gain in Faba Bean (Vicia Faba L.)
Source: Front Plant Sci. 2021 Oct 13;12:744259. doi: 10.3389/fpls.2021.744259 (PMC8548637; doi:10.3389/fpls.2021.744259)
Supplement: Supplementary file 1 [file Table_1.docx]

**Supplementary Table 1.** Major sources of resistance to biotic and abiotic stresses in faba bean.

| **Traits** | **Yield reduction (References)** | **Accessions** | **Origin** | **References** |
| --- | --- | --- | --- | --- |
| Ascochyta blight | 35 to 90% (Hampton, 1980; Diaz-Ruiz et al., 2009) | IG72433 | Syria | Maalouf et al. (2016) |
|  |  | G102181, IG105831, IG105860 | Spain |  |
|  |  | IG105863, IG105863, IG106583, Ascot | Morocco |  |
|  |  | Farah, Nura, PBA Rana, PBA Samira, PBA Amberley | Australia | Pulse Australia, 2021 |
| Chocolate spot | 50-90% (Gorfu and Yaynu, 2001; Beyene et al., 2018) | Moti, Obsie, Gora, Didea | Ethiopia | Maalouf et al. (2016) |
|  |  | IG102068, IG102468 | Turkey |  |
|  |  | ILB-4726, ILB-938, BPL-710 | ICARDA | Beyne et al., 2018 |
|  |  | Icarus, PBA Amberley | Ecuador | Pulse Australia (2021) |
| Rust | 30-68% (Marcellos et al., 1995; Rashid and Bernier, 1991) | Doza#12034, AC1655, Ac1231#14905, Ac1866#15013, Ac1269 (BPL 748), AC1272 (BPL 1179), PBA Nasma, PBA Warda, PBA Nanu | Australia | Adhikari et al. (2016); Ijaz et al. (2021) |
|  |  | 2N52 | Spain | Avila et al. (2003) |
|  |  | ILB3025, ILB3107, BPL7, BPL8, BPL260 | ICARDA | Herath et al., 2001 |
| Combined chocolate spot and rust | similar to above | IG102639 | Canada | Maalouf et al. (2016); Maalouf et al. (2019) |
|  |  | IG106583 | Columbia |  |
| Combined chocolate spot and Ascochyta blight |  | IG11562, IG12111 | Algeria | Maalouf et al. (2011) |
|  |  | IG11761 | Canada |  |
|  |  | IG102938 | Columbia |  |
|  |  | IG102230 | Lebanon |  |
|  |  | IG105862, IG105862, IG13777 | Morocco |  |
|  |  | IG105761, IG102180, IG102182, IG102182, IG105831, IG105762, IG105832 | Spain |  |
|  |  | IG11946 | Sweden |  |
|  |  | IG14115, IG12116, IG105847 | Tunisia |  |
| Multiple resistance to chocolate spot, Ascochyta blight and rust |  | IG101763 | Jordan | Maalouf et al. (2011) |
|  |  | IG104545 | Lebanon |  |
|  |  | IG105251, IG105303, IG105303, IG105473, IG105499 | Spain |  |
|  |  | IG105499, IG105612, IG105643, IG105696, IG105974, | Spain |  |
|  |  | IG106668 | Italy |  |
| Bean leaf roll virus | 70% (van Leur, 2021; pers. comm.) | AC1206#20033, Ac1210#20036, 13NF361#21021 | Australia | van Leur J., 2021, pers. comm. |
| Heat tolerance | 24-100% (Bishop et al., 2016) | Hudeiba93, Edamer, Basabeer | Sudan | Maalouf F., 2021, pers. Comm. |
|  |  | FB1197 | United Kingdom |  |
|  |  | FB1482 | Netherlands |  |
|  |  | FB2047 | Peru |  |
|  |  | FB2077 | France |  |
|  |  | IG11908 | Ethiopia |  |
|  |  | IG13771 | Morocco |  |
|  |  | VF683, VF729 | Spain |  |
| Salinity tolerance | 12-50% (Farooq et al., 2017) | Icarus | Australia | Tavakkoli et al. (2012); Farooq et al. (2017) |
| Acidic soil tolerance | - | Aurora | Sweden | Belachew et al. (2017) |
|  |  | Kassa, GLA 1103, Aurora, Messay, Dosha, NC 58 | Ethiopia |  |
| Combined tolerance to Imazethapyr and Metribuzin | 15-70% (Abou Khater et al., 2021) | VF335 | Russia | Abou Khater et al. (2021) |
|  |  | FB2568, FB2574 | France |  |
|  |  | IG132194 | China |  |
|  |  | FB1482 | Netherlands |  |
|  |  | IG12659 | Ethiopia |  |
|  |  | ILB1814 | Syria |  |
|  |  | Flip 86-98FB | Lebanon |  |
| Imidazolinone | 20-30% (Pulse Australia, 2021) | PBA Bendoc | Australia | Pulse Australia (2021) |
| Frost tolerance | 60% (Maqbool et al., 2010; Link et al., 2010) | Côte d’Or; Karl | France | Link et al. (2010) |
|  |  | Hiverna | Germany |  |
|  |  | BPL4628 | China |  |
|  |  | (Côte d’Or/1 x BPL4628)-95 | Göttingen, Germany |  |
|  |  | ILB14 | Syria |  |
|  |  | ILB345 | Egypt |  |
|  |  | ILB2999 | Pakistan |  |
|  |  | ILB3187 (Cixi Dabaican) | China |  |
|  |  | 11NF010a-2, PBA Warda, PBA Nasma, PBA Nanu | Australia | Alharbi et al. (2021) |
|  |  | ACV-42, ACV-84, ACV-88 | Turkey | Incy and Toker (2011) |
| Broomrape species | 37 to 90% (Abu-Irmaileh and Labrada, 2016) | Baraca, ILB4350, ILB4347 | Spain | Rubiales et al. (2016) |
|  |  | X-1714, X-1720, X-1671, Misr 1, Giza 843 | Egypt | Ashrie et al. (2010) |
|  |  | IG106670, IG106671, IG106672 | ICARDA/Morocco | Maalouf et al. (2011) |
|  |  | IG100187 | Egypt |  |
| Drought | 40-50% (Mwanamwenge et al., 1999) | DS70622 | Syria | Belachew et al. (2019) |
|  |  | DS11320 | Macedonia |  |
|  |  | ILB938/2 | Equador |  |

**References**

Abou-Khater, L., Maalouf, F., Patil, S. B., Balech, R., Nacouzi, D., Rubiales, D., et al. (2021). Identification of tolerance to metribuzin and imazethapyr herbicides in faba bean. *Crop Sci.* 61, 2593–2611. <https://doi.org/10.1002/csc2.20474>

Abu-Irmaileh, B. E., and Labrada, R. (2016). *The problem of Orobanche spp in Africa and Near East*. Available online at: <http://www.fao.org/agriculture/> crops/thematic-sitemap/theme/biodiversity/weeds/issues/oro/en/ (accessed on August 27, 2021).

Adhikari, K. N., Zhang, P., Sadeque, A., Hoxha, S., and Trethowan, R. (2016). Single independent genes confer resistance to faba bean rust (*Uromyces viciae- fabae*) in the current Australian cultivar Doza and a central European line Ac1655. *Crop Pasture Sci.* 67, 649–654. <https://doi.org/10.1071/CP15333>

Alharbi, N. H., Alghamdi, S. S., Migdadi, H. M., El-Harty, E. H., and Adhikari, K. N. (2021) Evaluation of frost damage and pod set in faba bean (*Vicia faba* L.) under field conditions. *Plants* 10, 1925 <https://doi.org/10.3390/plants10091925>

Ashrie, A. M. A., Eman, A. I., Helal, A. A., Abdel-Tawab, Y. M., and EL-Harty, E. H. (2010). Performance of six faba bean genotypes under free and *Orobanche* soils*. Egypt. J. Plant Breed.* 14, 189–205.

Avila, C. M., Sillero, J. C., Rubiales, D., Moreno, M. T., and Torres, A. M. (2003). Identification of RAPD markers linked to the Uvf-1 gene conferring hypersensitive resistance against rust (*Uromyces viciae-fabae*) in *Vicia faba* L. *Theor. Appl. Genet*. 107, 353–358. <https://doi.org/10.1007/s00122-003-1254-8>

Belachew, K. Y., and Stoddard, F. L. (2017). Screening of faba bean (*Vicia faba* L.) accessions to acidity and aluminium stresses. *PeerJ* 5, e2963 <https://doi.org/10.7717/peerj.2963>

Belachew, K. Y., Nagel, K. A., Poorter, H., and Stoddard, F. L. (2019). Association of shoot and root responses to water deficit in young faba bean (*Vicia faba* L.) plants. *Front. Plant Sci.* 10, 1063 <https://doi.org/10.3389/fpls.2019.01063>

Beyene, A. T., Derera, J., and Sibiya, J. (2018). Genetic variability of faba bean genotypes for chocolate spot (*Botrytis fabae*) resistance and yield. *Euphytica* 214, 132. <https://doi.org/10.1007/s10681-018-2210-7>

Bishop, J., Jones, H. E., O’Sullivan, D. M., and Potts, S. G. (2016). Elevated temperature drives a shift from selfing to outcrossing in the insect- pollinated legume, faba bean (*Vicia faba*). *J. Exp. Bot.* 68, 2055–2063. <https://doi.org/10.1093/jxb/erw430>

Díaz-Ruiz, R., Satovic, Z., Ávila, C. M., Alfaro, C. M., Gutierrez, M. V., Torres, A. M., et al. (2009). Confirmation of QTLs controlling *Ascochyta fabae* resistance in different generations of faba bean (*Vicia faba* L.). *Crop Pasture Sci.* 60, 353–361. <https://doi.org/10.1071/CP08190>

Farooq, M., Gogoi, N., Hussain, M., Barthakur, S., Paul, S., and Bharadwaj, N. (2017). Effects, tolerance mechanisms and management of salt stress in grain legumes. *Plant Physiol. Biochem.* 118, 199–217. <https://doi.org/10.1016/j.plaphy.2017.06.020>

Gorfu, D., and Yaynu, H. (2001). Yield loss of crops due to plant diseases in Ethiopia. *Pest Manag. J. Ethiopia* 5, 55–67.

Hampton, G. J. (1980). The significance of Ascochyta fabae in broad beans in the Manawatu, and methods for its control. *N. Z. J. Crop Hort. Sci.* 8, 305–308. <https://doi.org/10.1080/03015521.1980.10426279>

Herath, I. H. M. H. B., Stoddard, F. L., and Marshall, D. R. (2001). Evaluating faba beans for rust resistance using detached leaves. *Euphytica* 117, 47–57. <https://doi.org/10.1023/A:1004071916288>

Ijaz, U., Sudheesh, S., Kaur, S., Sadeque, A., Bariana, H., Bansal, U., et al. (2021). Mapping of two new rust resistance genes *Uvf-2* and *Uvf-3* in faba bean. *Agronomy* 11, 1370. <https://doi.org/10.3390/agronomy11071370>

Link, W., Balko, C., and Stoddard, F. L. (2010). Winter hardiness in faba bean: physiology and breeding. *Field Crops Res.* 115, 287–296. <https://doi.org/10.1016/j.fcr.2008.08.004>

Maalouf, F., Hu, J., O'Sullivan, D.M., Zong, X., Hamwieh, A., Kumar, S., and Baum, M. (2019). Breeding and genomics status in faba bean (*Vicia faba*). *Plant Breed.* 138, 465–473. <https://doi.org/10.1111/pbr.12644>

Maalouf, F. (2017). Developing improved varieties of faba bean. Burleigh Dodds Science.

Maalouf, F., Ahmed, S., Shaaban, K., Bassam, B., Nawar, F., Singh, M., and Amri, A. (2016). New faba bean germplasm with multiple resistances to Ascochyta blight, chocolate spot and rust diseases. *Euphytica* 211, 157–167. <https://doi.org/10.1007/s10681-016-1726-y>

Maalouf, F., Khalil, S., Ahmed, S., Kharrat, M., Hajjar, S., and El Shama’a, K. (2011). Yield stability of faba bean lines under diverse broomrape prone production environments. *Field Crops Res.* 124, 288–294. <https://doi.org/10.1016/j.fcr.2011.06.005>

Maqbool, A., Shafiq, S., and Lake, L. (2010). Radiant frost tolerance in pulse crops—a review. *Euphytica* 172, 1–12. <https://doi.org/10.1007/s10681-009-0031-4>

Marcellos, H., Moore, K. J., and Nikandrow, A. (1995). Influence of foliar-applied fungicides on seed yield of faba bean (*Vicia faba* L.) in northern New South Wales. *Aust. J. Exp. Agric.* 35, 97–102. <https://doi.org/10.1071/EA9950097>

Mwanamwenge, J., Loss, S. P., Siddique, K. H. M., and Cocks, P. S. (1999). Effect of water stress during floral initiation, flowering and podding on the growth and yield of faba bean (*Vicia faba* L.). *Eur. J. Agron.* 11, 1–11. <https://doi.org/10.1016/S1161-0301(99)00003-9>

Pulse Australia (2021). Available online at: https://[www.pulseaus.com.au/growing-](http://www.pulseaus.com.au/growing-) pulses/bmp/faba-and-broad-bean (accessed on August 27, 2021).

Rashid, K. Y., and Bernier, C. C. (1991). The effect of rust on yield of faba bean cultivars and slow-rusting populations. *Can. J. Plant Sci*. 71, 967–972. <https://doi.org/10.4141/cjps91-139>

Rubiales, D., Rojas-Molina, M. M., and Sillero, J. C. (2016). Characterization of Resistance Mechanisms in Faba Bean (*Vicia faba*) against Broomrape Species (*Orobanche* and *Phelipanche* spp.). *Front. Plant Sci.* 7, 1747. <https://doi.org/10.3389/fpls.2016.01747>

Tavakkoli, E., Paull, J., Rengasamy, P., and McDonald, G. K. (2012). Comparing genotypic variation in faba bean (*Vicia faba* L.) in response to salinity in hydroponic and field experiments. *Field Crops Res.* 127, 99–108. <https://doi.org/10.1016/j.fcr.2011.10.016>
